# LAG-3–associated CD8^+^ T-cell dysfunction in the cervical cancer tumor microenvironment

**DOI:** 10.3389/fimmu.2026.1750726

**Published:** 2026-01-28

**Authors:** Guang Zhang, Lei Wang, Jianhuan Chen, Jing Wang, Abidan Ainiwaer, Cailing Ma

**Affiliations:** 1 Department of Gynecology, The First Afffliated Hospital of Xinjiang Medical University, Urumqi, Xinjiang, China; 2 State Key Laboratory of Pathogenesis, Prevention and Treatment of High Incidence Diseases in Central Asia, Urumqi, Xinjiang, China; 3 Basic Medical College, Xinjiang Medical University, Urumqi, Xinjiang, China

**Keywords:** CD8+ T, cervical cancer, Immune checkpoint, LAG-3, tumor microenvironment(TME)

## Abstract

**Introduction:**

Cytotoxic T lymphocytes (CTLs, mainly CD8^+^ T cells) are the primary killer cells in the tumor microenvironment (TME). This study investigates the expression of LAG-3 in the TME of cervical cancer and its regulatory role in CD8^+^ T cell function.

**Methods:**

Cervical tissue pathological sections and fresh cervical tissues were collected from patients with cervical cancer, patients with high-grade squamous intraepithelial lesion (HSIL), and patients who underwent hysterectomy for non-cervical diseases (without a history of other tumors). Immunohistochemistry, Western blotting, quantitative real-time PCR, and fluorescence imaging techniques were used to analyze the expression of LAG-3 in the cervical cancer TME and its correlation with clinical tumor stage, differentiation grade, lymph node metastasis status, and lymphovascular space invasion. A cell co-culture system and a cervical cancer mouse model were established to evaluate the effects of lag-3 on tumor growth and changes in CD8^+^ T cell function.

**Results:**

LAG-3 is highly expressed in the tumor microenvironment (TME) of cervical cancer, with its expression level increasing as the tumor stage progresses: the lower the degree of differentiation, the higher the LAG-3 expression; LAG-3 expression is also elevated in cases with lymph node metastasis and lymphovascular space invasion. *In vivo* mouse models confirmed that lag-3 inhibits CD8^+^ T cells from expressing Ki67, T-bet, TNF-α, IFN-γ, and IL-2. Furthermore, we found that lag-3 not only suppresses the differentiation of naive CD8^+^ T cells into central memory T cells (TCM) but also inhibits the differentiation of TCM into effector memory T cells (TEM), a subset with stronger anti-tumor effector functions. A similar phenomenon was observed in cell co-culture experiments.

**Discussion:**

We confirmed that LAG-3 is highly expressed in cervical cancer tissues and is closely correlated with clinical stage, differentiation grade, lymph node metastasis, and lymphovascular space invasion. LAG-3 may inhibit the function of CD8^+^ T cells in the cervical cancer TME, thereby promoting the progression of cervical cancer.

## 1 Introduction

Cervical cancer remains a major cause of cancer-related mortality in women worldwide (1). Although originating in the cervix, it frequently invades the uterine corpus, parametrial tissues, vagina, and regional lymph nodes, profoundly compromising patient outcomes (2). A hallmark of cervical cancer progression is the dysfunction of T cells—particularly CD8^+^ T cells—driven in part by sustained expression of inhibitory immune checkpoints such as PD-1 and CTLA-4 (3).While blockade of PD-1/CTLA-4 has yielded clinical benefit in advanced disease, the role of lymphocyte activation gene 3 (LAG-3) in shaping antitumor immunity in cervical cancer has remained unclear (4). Recent studies have suggested that inhibitory receptors, including LAG-3, may contribute to CD8^+^ T-cell dysfunction in solid tumors (5). The causal link between human papillomavirus (HPV) infection and cervical cancer is well established (6), and the protective efficacy of HPV vaccination is widely recognized globally (7). Nevertheless, worldwide vaccination coverage remains far below the targets set by the World Health Organization (WHO), particularly in low- and middle-income countries, where cervical cancer incidence and mortality continue to rise (8). Epidemiological data indicate that in 2020, there were 604,127 new cases of cervical cancer worldwide, resulting in 341,831 deaths; by 2022, new cases had increased to 661,021, with 348,189 deaths (9). Cervical cancer thus remains one of the leading causes of cancer-related mortality among women, and its global disease burden continues to escalate.

Current standard-of-care treatments for cervical cancer primarily involve surgery combined with radiotherapy and chemotherapy. While these interventions achieve partial clinical efficacy, they are associated with substantial morbidity (10). Extensive surgical resection profoundly impacts quality of life, whereas radiotherapy and chemotherapy frequently induce severe adverse effects, including bone marrow suppression, anemia, gastrointestinal toxicity, ototoxicity, nephrotoxicity, neuropathic pain, Raynaud’s phenomenon, enteritis, and cystitis (11). In recent years, the advent of immune checkpoint inhibitors (ICIs) has partially mitigated the limitations of conventional therapies, highlighting the therapeutic potential of modulating the functional state of immune cells within the tumor immune microenvironment (TIME) (12).

CD8^+^ T cells are central mediators of antitumor immunity in cervical cancer (13). Compared with normal cervical tissue, cervical cancer lesions exhibit markedly increased infiltration of CD8^+^ T cells (14). Paradoxically, despite this high density, the cytotoxic activity of these T cells is often impaired, implying that immunosuppressive factors within the TIME restrict their function. Indeed, elevated PD-L1 expression has been reported in cervical cancer and correlates strongly with lymph node metastasis (15, 16). PD-L1 engagement of PD-1 on T cells profoundly suppresses CD8^+^ T-cell effector function, thereby promoting immune evasion, and PD-L1 blockade partially restores T-cell activity and restrains tumor progression (17). Beyond PD-1/PD-L1, additional inhibitory checkpoints—including CTLA-4, LAG-3, TIGIT, TIM-3, and IDO—have been shown to compromise CD8^+^ T-cell function across multiple malignancies, thereby facilitating tumor development and progression (18–20). Consequently, immune checkpoints are among the most promising therapeutic targets in cancer immunotherapy.

Lymphocyte-activation gene 3 (LAG-3, CD223) is a novel inhibitory checkpoint broadly expressed on activated CD4^+^ and CD8^+^ T cells. Under chronic antigen stimulation, such as within tumors, LAG-3 expression is sustained, driving T cells toward an exhausted phenotype (21). Mechanistically, LAG-3 primarily mediates immunosuppression via binding to major histocompatibility complex class II (MHC-II) molecules, thereby inhibiting T-cell proliferation and effector function (22). Additionally, LAG-3 can engage alternative ligands, including fibrinogen-like protein 1 (FGL1), and influence tumor biology through mechanisms such as epithelial–mesenchymal transition (EMT), epigenetic reprogramming, oxidative metabolism, and lipid metabolism (23). High LAG-3 expression has been observed in diverse tumors, where it directly or indirectly suppresses CD8^+^ T-cell differentiation and cytotoxic function, facilitating immune escape. Preclinical studies show that the LAG-3-blocking antibody relatlimab enhances T-cell receptor (TCR) signaling, promotes CD8^+^ T-cell differentiation, and increases cytotoxicity (24). Furthermore, lag-3-deficient CD8^+^ T cells display broader TCR clonality, enrichment of effector- and interferon-response–related gene signatures, and elevated IFN-γ secretion, collectively reflecting potentiated antitumor immunity (25). These findings highlight the critical need to elucidate how LAG-3 modulates CD8^+^ T-cell function within the cervical cancer TIME and its contribution to disease progression.

In this study, we systematically investigated the role of LAG-3 in regulating CD8^+^ T-cell function during cervical cancer progression. We analyzed tissue specimens from 60 patients with pathologically confirmed cervical cancer, 60 patients with high-grade squamous intraepithelial lesions (HSIL), and 60 normal cervical tissues obtained from hysterectomies performed for non-cervical indications. All patients had no prior history of malignancy. Using immunohistochemistry, immunoblotting, and multiplex immunofluorescence, we found that LAG-3 was significantly upregulated in cervical cancer tissues and predominantly localized to CD8^+^ T cells. In *in vitro* co-culture assays, lag-3-deficient CD8^+^ T cells retained robust effector activity and exhibited markedly increased cytokine production, with IL-2 and TNF-α levels significantly elevated relative to their lag-3-expressing counterparts. Consistently, in the *in vivo* subcutaneous cervical cancer model, lag-3-deficient mice showed enhanced CD8^+^ T-cell functionality—characterized by augmented cytotoxic responses—and a substantially reduced tumor growth rate, underscoring the cell-intrinsic inhibitory role of LAG-3 in anti-tumor immunity.

## 2 Materials and methods

### 2.1 Human subjects

Pathological Tissue Specimens:A total of 180 formalin-fixed, paraffin-embedded (FFPE) cervical tissue specimens were collected from patients at Xinjiang Medical University between January 2019 and December 2022. These included: 60 cases of histopathologically confirmed cervical cancer, 60 cases of high-grade cervical intraepithelial neoplasia (HSIL), and 60 cases of normal cervical tissue obtained from patients undergoing hysterectomy for benign uterine or adnexal conditions unrelated to malignancy. All enrolled patients had no prior history of hypertension, diabetes mellitus, or other malignancies. Tissue specimens were obtained from the Department of Pathology, Xinjiang Medical University. Cervical Cancer and Normal Cervical Tissue: Cervical cancer and normal cervical tissue samples were procured from the Obstetrics and Gynecology Center of Xinjiang Medical University. All patients provided written informed consent prior to surgery. The collection and subsequent use of these specimens were approved by the institutional ethics committee, ensuring compliance with medical ethics and patient privacy protection standards. All sample collection and usage strictly adhered to the ethical guidelines and standardized operating procedures of the ethics committee of the First Affiliated Hospital of Xinjiang Medical University (A240522-90).

### 2.2 Cell lines

The U14 murine cervical cancer cell line used in this study was purchased from Wuhan Boshi Biotechnology Co., Ltd. The cells were authenticated and confirmed to meet experimental quality standards prior to use.

### 2.3 Animal models

Female C57BL/6 mice, aged 8~10 weeks and weighing 20 ± 2 g, were obtained from the Experimental Animal Center of Xinjiang Medical University. Female lag-3-deficient (lag3^-^/^-^) mice of the same age and weight were purchased from Shanghai Southern Model Biotechnology Co., Ltd. All animals were maintained under specific pathogen-free (SPF) conditions in the Xinjiang Medical University Experimental Animal Center, following standard protocols for laboratory animal care and handling. All animal experiments were conducted in accordance with institutional guidelines and were approved by the ethics committee of the First Affiliated Hospital of Xinjiang Medical University (A240522-90).

### 2.4 Instruments and reagents

Confocal imaging was performed using a ZEN series laser-scanning confocal microscope (Carl Zeiss AG, Germany). Flow cytometric analyses were conducted on instruments provided by Becton, Dickinson and Company (BD, USA). Cell culture reagents, including RPMI-1640, DMEM, and PBS, were purchased from HyClone (USA). Cell stimulants were obtained from eBioscience (USA). Red blood cell lysis buffer, cytokine fixation and permeabilization buffers, and anti-mouse Fc receptor blocking antibody (α-CD16/32) were purchased from BioLegend (USA). LAG-3 antibodies for immunohistochemistry (IHC) were obtained from Proteintech (USA). Kits for isolation of mouse peripheral blood mononuclear cells (PBMCs) and tumor-infiltrating lymphocytes (TILs) were obtained from Solarbio (China). Multiplex fluorescence immunohistochemistry (mIHC) was performed using a six-color TSA staining kit (PDOne Six-Color TSA-RM-82758, 50T) from Panoramic Biotech (China). Flow cytometry antibodies, including NK1.1, CD3, CD4, CD8, CD44, CD62L, CD69, Foxp3, LAG-3, Ki-67, T-bet, IL-10 IFN-γ, TNF-α, and IL-2, were purchased from BioLegend (USA) and validated for flow cytometry applications.

### 2.5 Immunohistochemistry

FFPE tissue sections were baked at 60°C for 2 hours, followed by standard deparaffinization and graded ethanol rehydration. Antigen retrieval was performed in Tris-EDTA buffer via heat-mediated epitope exposure. Sections were blocked with 10% goat serum in TBST for 30 minutes at room temperature, after which primary antibody against LAG-3 (rabbit polyclonal, 1:750 dilution) was applied and incubated at 4°C for 8 hours. After returning to room temperature and equilibration for 1 hour, sections were incubated with HRP-conjugated secondary antibody (goat anti-rabbit, 1:500) for 2 hours at room temperature. Signal detection was achieved using DAB chromogen, and nuclei were counterstained with hematoxylin. Sections were differentiated for 1–2 seconds, blued for 2 minutes (adjusted as needed), dehydrated through graded ethanol, cleared in xylene, and mounted with neutral resin. LAG-3 expression distribution and the proportion of positive cells were evaluated under light microscopy across different cervical pathological types.

### 2.6 Multiplex immunofluorescence

Paraffin sections were baked at 60°C for 2 hours, deparaffinized, rehydrated, and subjected to heat-induced antigen retrieval in citrate buffer. Sections were blocked for 30 minutes at room temperature, then incubated with primary antibody against CD4 cells overnight at 4°C. Fluorescently labeled secondary antibodies were applied for 10 minutes, followed by TSA signal amplification with fluorescent dyes, adjusting volume according to tissue area. Sequential rounds of heat-induced antigen retrieval and blocking were performed to stain CD4, CD8, and LAG-3. CD8 and LAG-3 antibodies were incubated overnight at 4°C. DAPI was used for nuclear staining, and sections were mounted with anti-fade medium. Imaging was performed using a ZEN 3.4 confocal microscope (blue edition), capturing co-localization of CD4, and CD8 cells with LAG-3, and quantitative image analysis was performed.

### 2.7 Western blotting

Cervical cancer and normal cervical tissues were minced and lysed in RIPA buffer supplemented with PMSF in 2 mL EP tubes. Lysates were sonicated and centrifuged at 12,000 rpm for 5 minutes at 4°C. Supernatants were collected as protein samples. Protein concentrations were determined using a BCA assay. Appropriate polyacrylamide gels were prepared according to target protein molecular weights, and proteins were separated via SDS-PAGE and transferred onto PVDF membranes. Membranes were blocked with 5% non-fat milk for 2 hours, incubated with primary antibody against LAG-3 overnight at 4°C, washed with TBST, and incubated with HRP-conjugated secondary antibody for 1 hour at room temperature. Protein bands were visualized using ECL chemiluminescence and quantified with Image Lab software.

### 2.8 Murine tumor model establishment

Five female C57BL/6 wild-type (WT) and five lag-3-knockout (lag3-KO) mice (8~10 weeks old, 20 ± 2 g) were subcutaneously inoculated with 1×10^7^ U14 cells in 200 µL PBS in the right axillary region. Tumor growth was monitored daily for 2 weeks, and tumors with a diameter ≥5 mm were considered successfully established.

### 2.9 Isolation of splenic lymphocytes

Prior to euthanasia, blood was collected via orbital puncture. Mice were euthanized by cervical dislocation, and spleens were harvested under sterile conditions. Spleens were gently dissociated on a glass slide in a culture dish, filtered through a 200-mesh nylon strainer, and centrifuged at 2,000 rpm for 5 minutes. The pellet was resuspended in 3 mL red blood cell lysis buffer and incubated on ice for 12 minutes. Lysis was terminated with PBS, followed by centrifugation and resuspension in 3 mL PBS. The suspension was filtered again, adjusted to 8 mL, and used for cell counting and flow cytometric analyses.

### 2.10 Isolation of tumor-infiltrating lymphocytes from subcutaneous tumors

Subcutaneous tumors were carefully excised post-mortem, minced into small fragments, and transferred into 15 mL centrifuge tubes containing digestion buffer (RPMI-1640 supplemented with 10% FBS, 0.2% collagenase IV, 0.01% hyaluronidase, and 0.002% DNase I). Tissues were digested on a 37°C orbital shaker for 90 min. The resulting cell suspension was filtered through a cell strainer and centrifuged at 2,000 rpm for 5 min. The cell pellet was resuspended to a final concentration of 1×10^8^ cells/mL. For density gradient separation, 3 mL of tumor lymphocyte separation medium was placed in a 15 mL tube tilted at 45°, and 3 mL of the single-cell suspension was carefully layered atop. Centrifugation was performed at 750 g, 20°C, for 30 min (acceleration 9, deceleration 9). The mononuclear cell layer was collected, washed with PBS, centrifuged, and resuspended to 3 mL for subsequent cell counting and flow cytometry analysis.

### 2.11 Phenotypic analysis of lymphocytes

One million (1×10^6^) lymphocytes were washed with PBS and centrifuged at 6,000 rpm for 2 min. The cell pellet was resuspended in Fc receptor blocking antibody (α-CD16/32) and incubated at 4°C for 30 min. Fluorochrome-conjugated antibodies against NK1.1, CD3, CD4, CD8, CD69, CD44, CD62L, and LAG-3 were added and incubated for 30 min in the dark. Cells were washed with PBS, resuspended in 300 µL PBS, filtered, and analyzed using flow cytometry.

### 2.12 Transcription factor analysis

Two million (2×10^6^) lymphocytes were washed with PBS, blocked with α-CD16/32 for 30 min, and stained with surface markers (NK1.1, CD3, CD4, CD8), for 30 min at room temperature in the dark. After washing, cells were fixed with 200 µL fixation buffer for 30 min, centrifuged at 10,000 rpm for 3 min, and permeabilized twice with 500 µL permeabilization buffer. Intracellular staining with anti-Ki-67 and anti-T-bet antibodies was performed for 45 min at room temperature in the dark. Following PBS washes, cells were resuspended in 300 µL PBS, filtered, and analyzed by flow cytometry.

### 2.13 Cytokine secretion assay

Two million (2×10^6^) lymphocytes were washed with PBS and resuspended in complete RPMI-1640 containing 1× cell stimulation cocktail. Cells were incubated at 37°C in a 5% CO_2_ incubator for 4 hours. Post-stimulation, Fc receptors were blocked with α-CD16/32 for 30 min, followed by surface staining for NK1.1, CD3, CD4, CD8, and CD44 for 30 min in the dark. Cells were fixed with 200 µL fixation buffer for 30 min at room temperature, permeabilized twice, and stained with intracellular cytokine antibodies (IFN-γ, TNF-α, IL-2,IL-10) for 45 min in the dark. Cells were washed, resuspended in 300 µL PBS, filtered, and subjected to flow cytometric analysis to assess CD8^+^ T cell cytokine production.

### 2.14 Sorting of lag-3^-^ and lag-3^+^ CD8^+^ T cells (Magnetic Bead–Based Sorting)

Splenic lymphocytes were isolated from C57BL/6 wild-type (WT) and lag-3-knockout (lag-3^-^/^-^) mice by mechanical dissociation of spleens followed by red blood cell lysis using ACK lysis buffer. Cell suspensions were filtered through a 70-μm nylon mesh, washed twice with cold MACS buffer (PBS supplemented with 0.5% bovine serum albumin and 2 mM EDTA), and counted before magnetic separation.CD8^+^ T cells were first enriched using a negative selection strategy with a mouse CD8^+^ T Cell Isolation Kit (Miltenyi Biotec), according to the manufacturer’s instructions, to avoid unintended activation. Briefly, splenocytes were incubated with a biotin-conjugated antibody cocktail targeting non-CD8^+^ lineage cells, followed by anti-biotin microbeads. Labeled cells were depleted using LS columns placed in a MACS Separator (Miltenyi Biotec), and the unlabeled fraction containing enriched CD8^+^ T cells was collected.

For separation of lag-3^+^ and lag-3^-^ CD8^+^ T cells, the enriched CD8^+^ T-cell fraction was subsequently incubated with a biotin-conjugated anti-mouse lag-3 antibody (clone C9B7W, Miltenyi Biotec) for 15 min at 4°C, followed by labeling with anti-biotin microbeads for an additional 15 min. Cells were washed and applied to fresh LS columns in the magnetic field. lag-3^+^ CD8^+^ T cells were retained in the column and eluted after removal from the magnetic field, whereas the flow-through fraction was collected as lag-3^-^ CD8^+^ T cells. CD8^+^ T cells from lag-3^-^/^-^ mice were processed in parallel and served as lag-3-negative controls. The purity of lag-3^+^ and lag-3^-^ CD8^+^ T-cell populations was assessed by post-sort flow cytometric analysis using antibodies against CD3ϵ (clone 145-2C11), CD8α (clone 53-6.7), and lag-3 (clone C9B7W). The purity of both populations consistently exceeded 90–95%. Sorted cells were immediately used for downstream functional assays, including co-culture experiments and cytokine analyses, or cultured as indicated.

### 2.15 Co-culture of lag-3^-^/^+^ CD8^+^ T cells with U14 cells

A Transwell co-culture system (6-well plate) was employed. U14 cells were seeded in the upper chamber at a density optimized for experimental conditions in 3 mL complete medium, ensuring the medium exceeded the membrane by ~5 mm. Upon reaching ~40% confluency, 5×10^5^ lag-3^-^ or lag-3^+^ CD8^+^ T cells were added to the lower chamber. Co-culture proceeded for 24 h without medium replacement. After incubation, floating U14 cells in the lag-3^-^CD8^+^ T co-culture group indicated cell apoptosis or lysis. Lymphocytes were harvested from the lower chamber for subsequent functional assays, including flow cytometry as described above. (Note: Lymphocytes were isolated from the spleens of live mice.).

### 2.16 Statistical analysis

Data analyses and graphical presentations were performed using GraphPad Prism 10.1.2. Quantitative data are presented as mean ± SD. Comparisons between two groups were conducted using two-tailed unpaired t-tests (α = 0.05). For multiple-group comparisons, one-way ANOVA was applied with the same significance threshold. Fluorescence image quantification was performed using ZEN 3.4 (blue edition) and ImageJ software. Flow cytometry data were analyzed using FlowJo v10.8.1, and Western blot band intensities were quantified with Image Lab software.

## 3 Results

### 3.1 Expression profile of LAG-3 across cervical cancer sample types and CD8^+^ T-cell subsets

**Figure 1:**
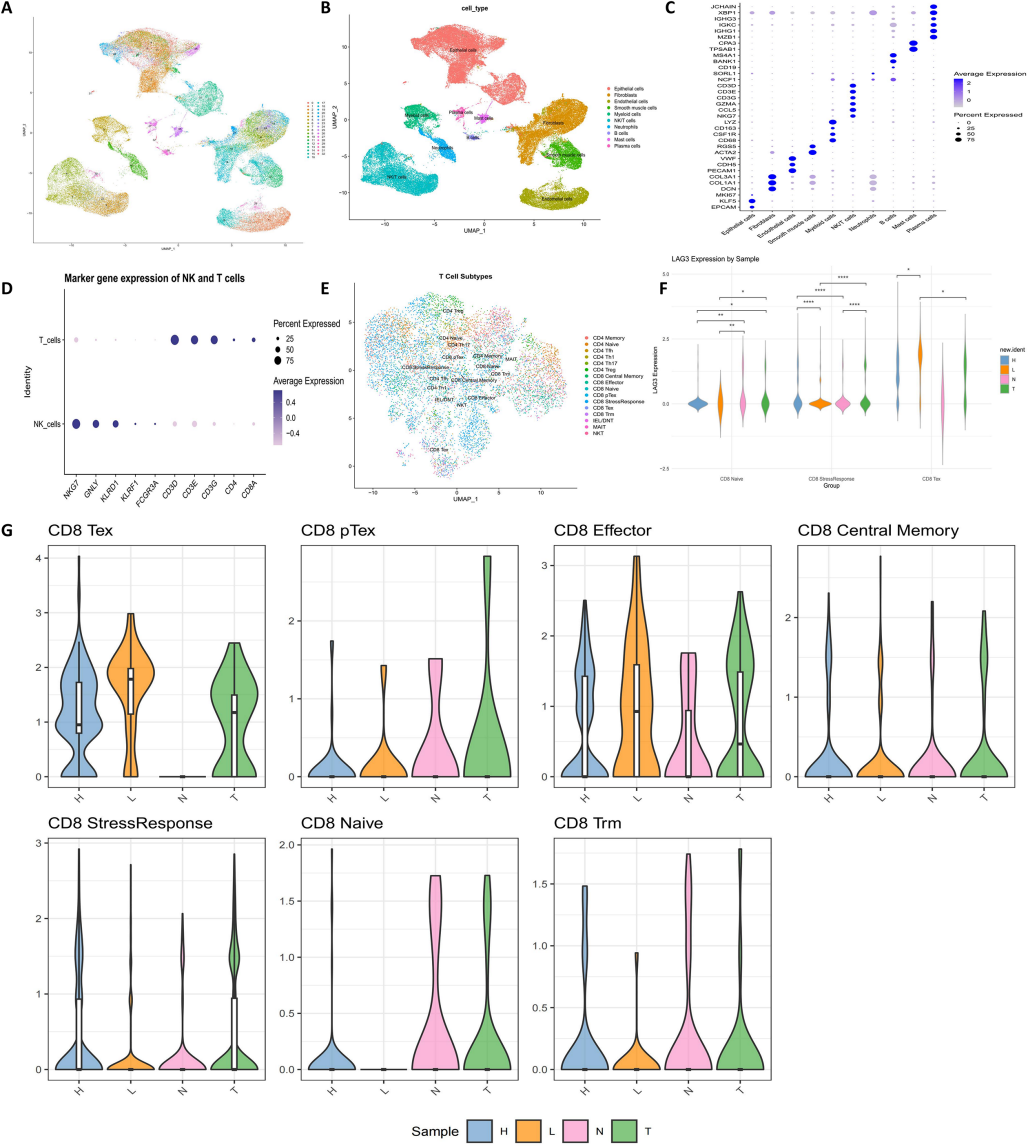
Single-cell atlas reveals cellular heterogeneity and LAG-3 expression dynamics in cervical cancer. **(A)** UMAP projection of single-cell transcriptomes after Harmony-based batch correction and PCA dimensionality reduction identified 33 transcriptionally distinct clusters, reflecting pronounced intratumoral heterogeneity. **(B)** Cell type annotation using canonical lineage markers categorized these clusters into 10 major cellular compartments within the cervical tumor microenvironment. **(C)** Distribution of cells by sample origin showed distinct spatial segregation across normal cervix (N), HSIL (H), primary tumor (T), and metastatic lymph node (L) in the integrated UMAP landscape. **(D)** Within the immune compartment, NK/T cells were delineated and T cell subsets were further resolved, enabling refined dissection of T cell heterogeneity. **(E)** Isolation of CD8^+^ T cells provided the basis for focused analyses of cytotoxic lymphocyte states across disease stages. **(F)** Cross-sample comparison revealed that LAG-3 expression was markedly elevated in CD8^+^ T cells from primary tumors (T) and metastatic lymph nodes (L) relative to normal cervical tissues (N). **(G)** LAG-3 exhibited a subset- and context-specific expression pattern, being preferentially enriched in exhausted T cells (Tex) and displaying distinct distribution across clinical sample types.

To characterize the dynamics of LAG-3 expression during cervical cancer progression, we analyzed single-cell transcriptomic data from the E-MTAB-12305 dataset, which included normal cervical tissues (N), high-grade squamous intraepithelial lesions (HSIL, H), primary cervical tumors (T), and metastatic lymph nodes (L). Following stringent quality control, 83,129 high-quality cells were retained for downstream analysis.To mitigate inter-sample variability, the Harmony algorithm was applied for batch-effect correction, enhancing cluster consistency while.

preserving genuine biological differences. Subsequent dimensionality reduction using principal component analysis (PCA) and UMAP visualization delineated 33 transcriptionally distinct cell clusters, demonstrating substantial cellular heterogeneity (**Figure 1A**). Systematic annotation based on canonical marker genes identified ten major cell populations, including epithelial cells, fibroblasts, myeloid cells, and NK/T cells (**Figures 1B, C**). Within the NK/T-cell compartment (marked by NKG7, CCL5, CD3D, CD3E, CD3G), T cells were further extracted (CD3D/E/G, CD4, CD8A), and the CD8^+^ T-cell population was specifically delineated (**Figures 1D, E**).

LAG-3 transcripts were detectable across CD8^+^ T cells in all sample types, albeit with marked variation in abundance. Notably, LAG-3 expression was significantly elevated in primary tumors (T) and metastatic lymph nodes (L) relative to normal cervical tissue (N) (**Figure 1F**). Subclustering of CD8^+^ T cells provided higher-resolution insight into functional heterogeneity, revealing LAG-3 expression across defined T-cell subsets and disease stages (**Figure 1G**). LAG-3 was most highly expressed in CD8^+^ exhausted T cells (Tex), consistent with its role as a canonical marker of T-cell dysfunction. Considerable upregulation was also observed in CD8^+^ effector T cells and CD8^+^ stress response T cells, particularly within tumor (T) and metastatic lymph node (L) samples, indicating that even effector-phenotype T cells acquire functional suppression in response to tumor-associated stress. In contrast, LAG-3 expression.

remained low in CD8^+^ naïve T cells, central memory T cells (Tcm), and tissue-resident memory T cells (Trm). Stratification by sample type revealed a progressive increase in LAG-3 expression from normal tissue (N) to HSIL (H), primary tumors (T), and metastatic lesions (L), with HSIL samples exhibiting intermediate levels between non-malignant and malignant states. This gradient suggests that LAG-3 upregulation parallels both disease progression and functional impairment of CD8^+^ T cells. Collectively, these results indicate that LAG-3 is preferentially enriched in exhausted and stress-adapted CD8^+^ T cells within the cervical cancer tumor microenvironment, highlighting its role as a mechanistic/biomarker-associated marker of CD8^+^ T-cell dysfunction rather than a direct therapeutic target. Consistent with observations by Hirahara et al. in cervical cancer, we also found enriched populations of CD8^+^ T cells expressing inhibitory markers, including LAG-3, at the invasive tumor front, underscoring a conserved immunosuppressive signature in the tumor microenvironment.” (Hirahara et al., Br J Cancer 2024) (26).

### 3.2 Correlation of LAG-3 expression with functional states of CD8^+^ T cells

**Figure 2:**
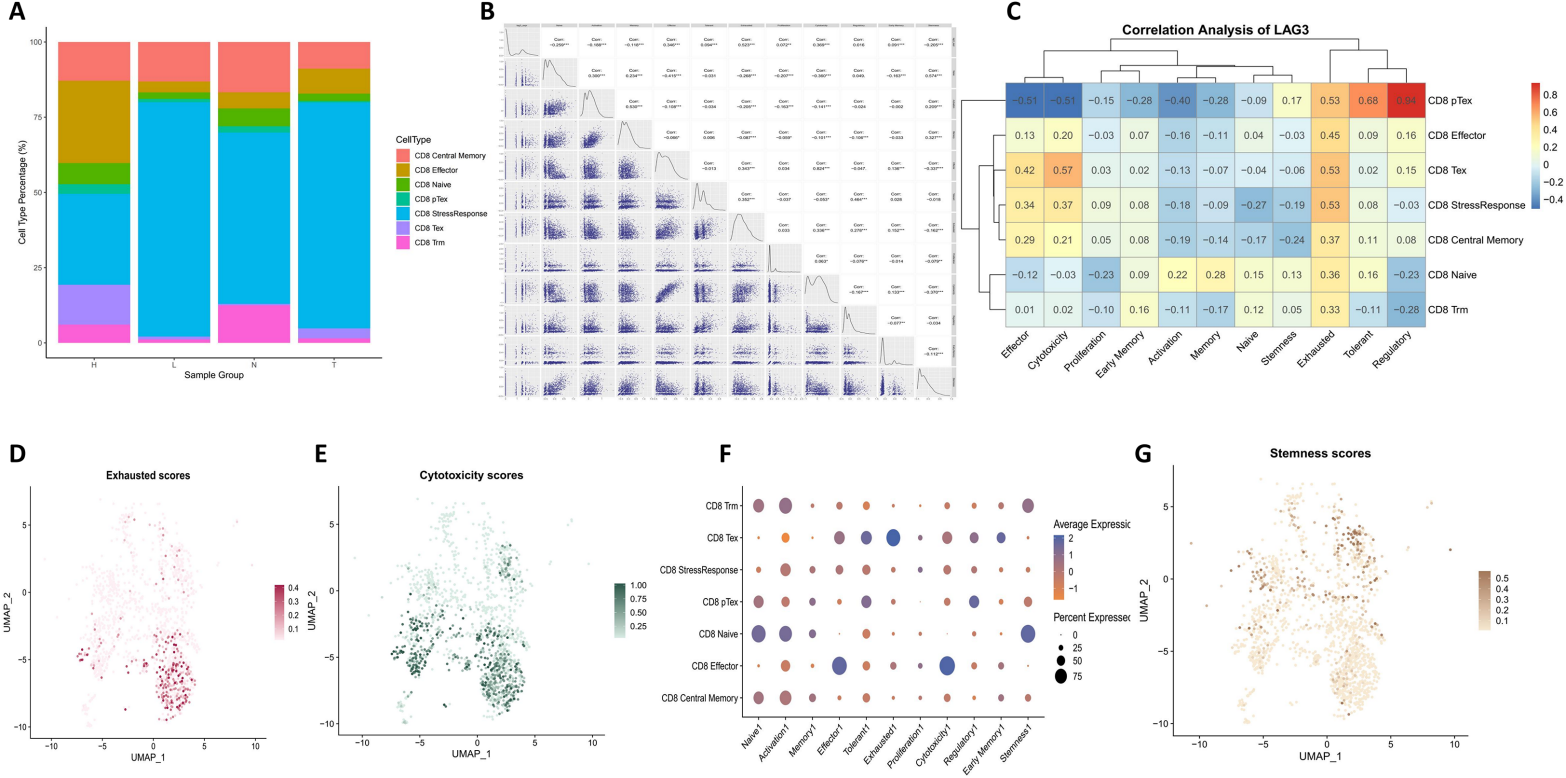
Functional landscape of CD8^+^ T cells and correlation with LAG-3 expression. **(A)** Distribution of CD8^+^ T-cell functional subsets across different pathological samples. The relative proportions of naïve, central memory, effector, stress-response, and exhausted CD8^+^ T-cell subsets are shown for normal cervix (N), high-grade squamous intraepithelial lesions (HSIL, H), and metastatic lymph nodes (L). **(B)** Correlation between LAG-3 expression and overall CD8^+^ T-cell functional scores. Pearson correlation analysis showing the relationship between LAG-3 transcript levels and scores for exhaustion, cytotoxicity, and effector function across the total CD8^+^ T-cell population. **(C)** Subset-specific correlation of LAG-3 expression with exhaustion scores. LAG-3 expression exhibits a strong positive correlation with exhaustion in CD8^+^ exhausted (Tex), effector, and stress-response T-cell subsets. **(D)** UMAP visualization of LAG-3 expression in CD8^+^ T cells. Each point represents a single cell, with color intensity reflecting relative LAG-3 transcript abundance. **(E)** UMAP projection of exhaustion scores within the CD8^+^ T-cell compartment. Color intensity indicates the functional exhaustion score for each cell, highlighting spatial enrichment of exhausted phenotypes. **(F)** UMAP visualization of cytotoxicity scores in CD8^+^ T cells. Cells are colored according to their cytotoxicity module score, revealing spatial patterns of effector potential. **(G)** UMAP projection of stemness scores in CD8^+^ T cells. Relative stemness scores are mapped onto the CD8^+^ T-cell population, showing enrichment of stem-like features in naïve subsets and their spatial separation from LAG-3-high exhausted cells.

As shown in **Figure 2A**, the distribution of CD8^+^ T cell subsets varied markedly across pathological conditions. In normal cervical tissue (N), the compartment was predominantly composed of central memory CD8^+^ T cells (CD8 TCM) and tissue-resident memory CD8^+^ T cells (CD8 Trm), which sustain long-term immune surveillance and rapid recall capacity. In high-grade squamous intraepithelial lesions (HSIL, H), the proportion of cytotoxic effector CD8^+^ T cells (CD8 Effector) and stress-adapted CD8^+^ T cells (CD8 StressResponse) was markedly increased, indicating activation of antitumor immunity while simultaneously reflecting microenvironmental pressure. By contrast, in metastatic lymph nodes (L), CD8 StressResponse cells became the dominant population, suggesting that the profoundly immunosuppressive milieu severely compromises CD8^+^ T cell functionality. To interrogate the potential functional role of LAG-3 in CD8^+^ T cells, we systematically assessed the correlation between its expression and predefined functional state signatures. Using the AddModuleScore algorithm, individual CD8^+^ T cells were scored for exhaustion, cytotoxicity, effector function, and stemness. At the global level, LAG-3 expression showed the strongest positive correlation with exhaustion scores (Pearson r = 0.523), followed by significant correlations with cytotoxicity (r = 0.369) and effector function (r = 0.346) (**Figure 2B**). Subset-specific analyses further revealed that in CD8 Tex, CD8 Effector, and CD8 StressResponse populations, LAG-3 expression remained tightly and positively correlated with exhaustion scores (**Figure 2C**).

These findings were reinforced by spatial mapping of functional scores: on the UMAP landscape, regions with high LAG-3 expression overlapped extensively with high exhaustion and high cytotoxicity scores (**Figures 2D–F**), delineating a transcriptional state in which LAG-3^+^ CD8^+^ T cells simultaneously exhibit exhaustion features while retaining residual effector potential. In contrast, stemness scores peaked in naïve CD8^+^ T cells, a compartment characterized by low LAG-3 expression (**Figure 2G**), suggesting that LAG-3 upregulation accompanies the exit from stem-like states during progressive differentiation. Collectively, these results position LAG-3 as a mechanistic/biomarker-associated marker of the exhausted trajectory in CD8^+^ T cells, supporting its relevance for understanding cellular dysfunction within the cervical cancer immune microenvironment rather than as a direct translational therapeutic claim (**Supplementary Figures 1**–**17**).

### 3.3 LAG-3 is highly expressed in human cervical cancer tissues and predominantly localized on CD8^+^ T cells

**Figure 3:**
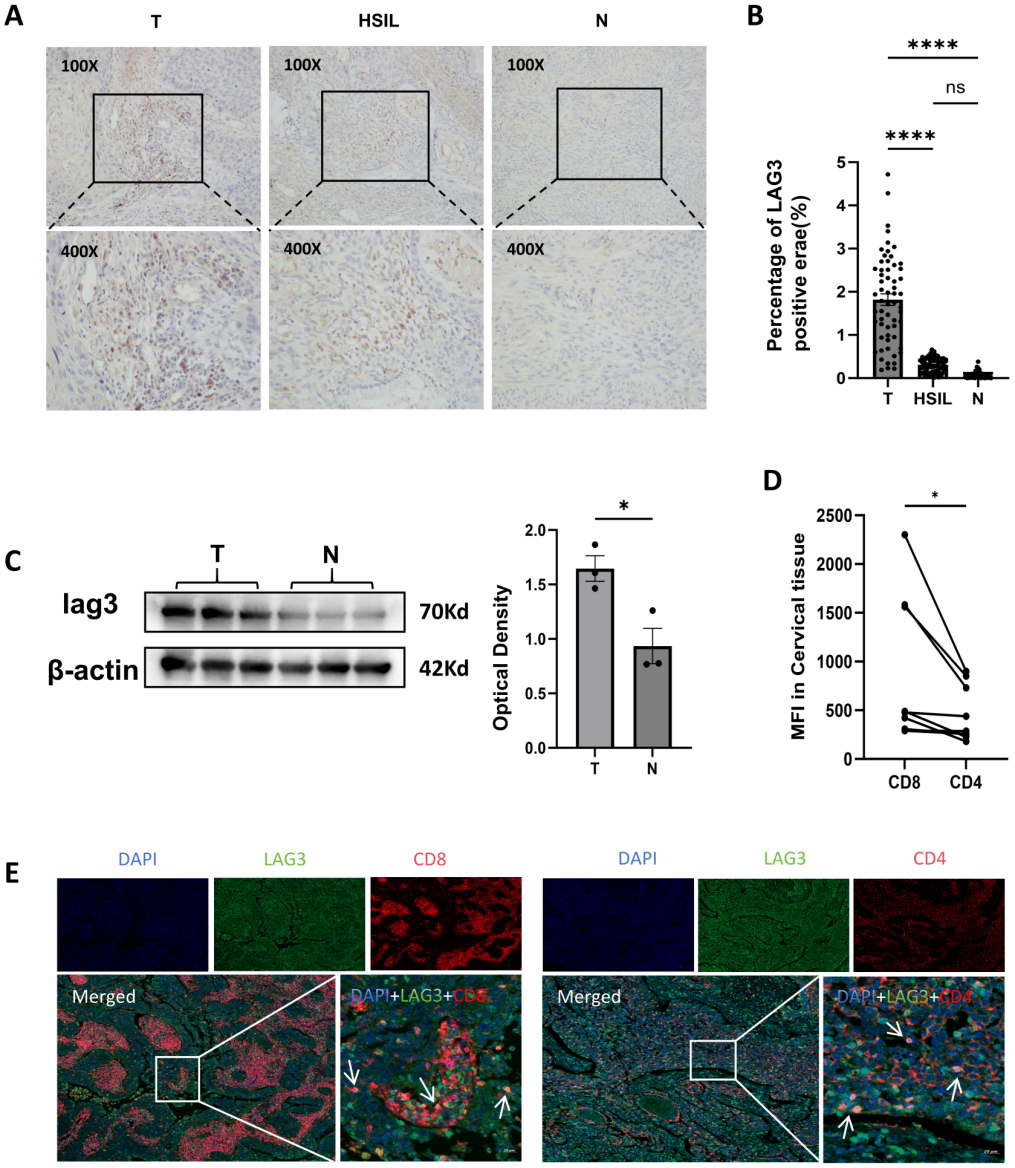
LAG-3 is upregulated in cervical cancer tissues and predominantly colocalizes with CD8^+^ T cells **(A)** Representative immunohistochemical staining of LAG-3 in cervical cancer tissues, high-grade squamous intraepithelial lesions (HSIL), and normal cervical tissues (upper panels, 100×; lower panels, 400×). **(B)** Quantitative analysis of LAG-3 expression across the three tissue groups, based on the percentage of positively stained area (n = 60 per group). **(C)** Western blot analysis of LAG-3 protein levels in cervical cancer versus normal cervical tissues, Semiquantitative assessment of LAG-3 protein expression based on optical density (OD) measurements. **(D)** Multiplex immunofluorescence staining (cervical cancer tissue sections) was performed to analyze the mean fluorescence intensity of CD4+ T cells and CD8+ T cells in the same field of view. (n=9) **(E)** Representative images of immunofluorescence co-staining of tissue sections from cervical cancer patients (n=9), showing 4’,6-diamidino-2-phenylindole (blue), CD8 or CD4 (red), LAG-3 (green), and merged images. The boxed area is the histological image at 200× magnification, and the arrows indicate CD8+LAG-3+ T cells or CD4+LAG-3+ T cells. All data are presented as mean ± SEM. *P < 0.05; ****P < 0.0001; n.s., not significant (P > 0.05). DAPI, 4′,6-diamidino-2-phenylindole.

To elucidate the expression pattern of LAG-3 in cervical cancer, we performed immunohistochemical (IHC) staining on cervical cancer tissues, high-grade squamous intraepithelial lesions (HSIL), and normal cervical tissues (**Figure 3A**). The results revealed robust LAG-3 expression within the tumor immune microenvironment of cervical cancer, primarily concentrated in peritumoral regions and broadly colocalized with infiltrating lymphocytes. Quantitative analysis across the three tissue types demonstrated that LAG-3 expression was significantly elevated in cervical cancer tissues compared to HSIL (P < 0.0001) and normal cervical tissues (P < 0.0001), whereas no statistically significant difference was observed between HSIL and normal cervix (P = 0.2212) (**Figure 3B**).

To further validate these observations, Western blot analysis was performed on cervical cancer and normal cervical tissues (**Figure 3C**). Consistent with IHC results, LAG-3 protein levels were markedly higher in cervical cancer tissues than in normal cervix (P = 0.024) (**Figure 3C**). Next, to investigate LAG-3 distribution among distinct lymphocyte subsets (CD8^+^ T cells and CD4^+^ T cells), multiplex immunofluorescence staining was conducted on representative cervical cancer sections. Nine histologically typical sections were selected based on hematoxylin and eosin (HE) staining and were stained for CD8^+^ and CD4^+^ Tcells, LAG-3, and nuclei (**Figure 3E**), Quantitative analysis of regional fluorescence intensity revealed that CD8^+^ T cells exhibited significantly higher mean fluorescence levels than CD4^+^ T cells (**Figure 3D**) consistent with prior reports by Hirahara et al. (*Br J Cancer*) demonstrating that tumor-infiltrating LAG-3^+^ lymphocytes in cervical cancer predominantly consist of CD8^+^ and CD4^+^ T cells (26). Similarly, Tsutsumi et al. (2025) reported markedly higher LAG-3 expression in cytotoxic CD8^+^ T cells (27).

In summary, LAG-3 is significantly highly expressed in cervical cancer tissues and preferentially distributed on CD8^+^ T cells, suggesting a potential mechanistic and biomarker-associated role in regulating CD8^+^ T-cell function and shaping the immune microenvironment, rather than directly predicting clinical outcomes or therapeutic response.

### 3.4 Association between LAG-3 expression and clinicopathological features and survival

Following the characterization of LAG-3 expression patterns, we further evaluated the clinical relevance of LAG-3 in cervical cancer. Patients were stratified into LAG-3 low (≤50%) and high (>50%) expression groups based on immunohistochemical positivity. Kaplan–Meier survival analysis revealed no significant difference in overall survival between the two groups (log-rank test, P > 0.05). Consistently, univariate Cox proportional hazards regression analysis did not identify LAG-3 expression as an independent prognostic factor for survival (HR ≈ 1, P > 0.05) (**Supplementary Table 6**, **Supplementary Figure 29**).

Notably, high LAG-3 expression was significantly associated with advanced tumor stage and poor differentiation; however, these associations did not translate into a statistically significant survival difference in our cohort. This may reflect the multifactorial determinants of survival in cervical cancer, as well as the relatively limited sample size and follow-up duration. Collectively, these findings suggest that LAG-3 expression may primarily reflect an immunosuppressive or exhausted T-cell phenotype associated with tumor progression, rather than serving as a direct determinant of long-term survival.

### 3.5 Correlation between LAG-3 expression and clinicopathological features of cervical cancer

We assessed LAG-3 expression by immunohistochemistry in pathological specimens from 60 patients who underwent surgical resection for cervical cancer and examined its association with key clinicopathological parameters, including tumor stage, histological differentiation, lymph node metastasis, and lymphovascular or perineural invasion. Among the 60 cases, squamous cell carcinoma accounted for 85%, adenocarcinoma for 13%, and adenosquamous carcinoma for a single case. Analysis by FIGO stage revealed that the percentage of LAG-3-positive areas was highest in patients with stage III C1 disease (2.906 ± 1.20) and lowest in stage IA patients (0.264 ± 0.07) (**Table 1**). Pairwise comparisons showed no statistically significant differences between stages IA and IB (P = 0.4756), nor between stages IIA1 and IIA2 (P = 0.4938) (**Table 2**). Despite the lack of significance between some early-stage subgroups, an overall trend was evident: LAG-3 expression progressively increased with advancing tumor stage.

**Table 1:** Clinicopathological characteristics of 60 surgically treated cervical cancer patients, stratified by FIGO stage, histological differentiation, lymph node metastasis, and lymphovascular/perineural invasion.

Parameter	Category	No. of patients	Immunohistochemical score (IHC score) ± SD
Pathological type	Squamous cell carcinoma (SCC)	51	
	Adenocarcinoma (ADC)	8	
	Adenosquamous carcinoma (ASC)	1	
FIGO stage	IA	5	0.264± 0.07
	IB	13	0.791± 0.25
	IIA1	20	2.003± 0.58
	IIA2	12	2.363± 0.59
	IIIC1	10	2.906± 1.02
Histological differentiation	Well-differentiated	7	0.841± 0.84
	Moderately to moderately–well differentiated	34	1.741± 0.90
	Poorly to moderately–poorly differentiated	19	2.316± 1.05
Lymph node metastasis	Present	10	2.906± 1.02
	Absent	50	1.600± 0.89
Lymphovascular and perineural invasion	Present	24	2.274± 1.02
	Absent	36	1.514± 0.92

**Table 2:** Statistical analysis of 60 surgically treated cervical cancer patients stratified by FIGO stage, histological differentiation, lymph node involvement, and lymphovascular/neural invasion.

Parameter	Comparison	Summary	P value
FIGO stage	IA vs IB	ns	0.4756
	IA vs IIA1	****	<0.0001
	IA vs IIA2	****	<0.0001
	IA vs IIIC1	****	<0.0001
	IB vs IIA1	****	<0.0001
	IB vs IIA2	****	<0.0001
	IB vs IIIC1	****	<0.0001
	IIA1 vs IIA2	ns	0.4938
	IIA1 vs IIIC1	***	0.0030
	IIA2 vs IIIC1	ns	0.2418
Histological Differentiation	G1 vs G2	ns	0.0642
	G1 vs G3	**	0.0023
	G2 vs G3	*	0.0924
Lymph Node Metastasis	YES vs no	**	0.0001
Lymphovascular and Perineural Invasion	YES vs no	*	0.0041

**Figure 4:**
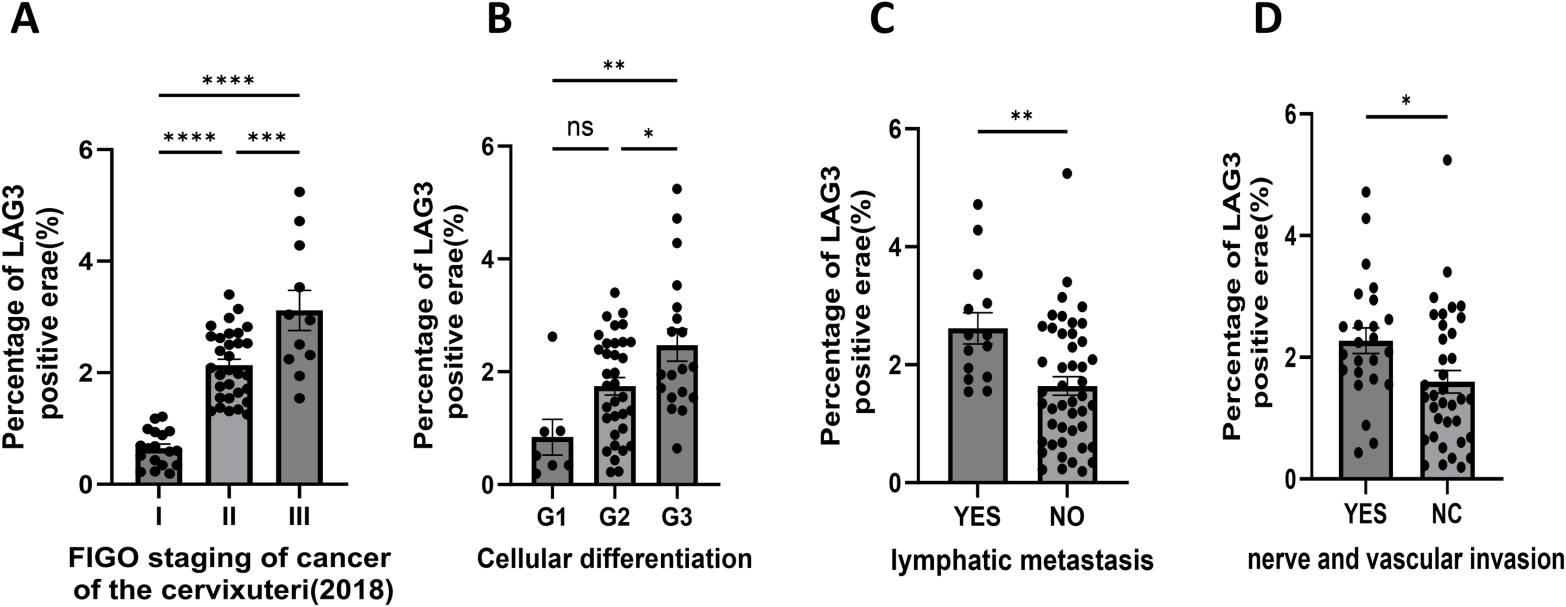
Statistical analyses of LAG-3 expression across key clinicopathological features of cervical cancer. **(A)** Comparison of LAG-3-positive expression among patient groups stratified by FIGO stage. **(B)** Comparison of LAG-3 expression across groups stratified by histological differentiation. **(C)** Comparison of LAG-3 expression between patients with and without lymph node metastasis. **(D)** Comparison of LAG-3 expression between patients with and without lymphovascular or perineural invasion. *p < 0.05; **p < 0.01; ***p < 0.001; ****p < 0.0001; ns, not significant.

Further stratification of patients by FIGO stage revealed that LAG-3-positive expression differed significantly across stage I, II, and III groups (P < 0.05, **Figure 4A**). Regarding histological differentiation, LAG-3 expression exhibited a negative correlation with differentiation status, such that lower differentiation was associated with higher LAG-3 levels. Specifically, the proportion of LAG-3-positive areas was 0.841 ± 0.84 in the well-differentiated group, 1.741 ± 0.90 in the moderately differentiated group, and 2.316 ± 1.05 in the poorly differentiated group; A significant difference was detected between the well-differentiated and poorly differentiated groups, whereas no significant differences were observed between the well- and moderately differentiated groups or between the moderately and poorly differentiated groups. (**Figure 4B**). When stratified by lymph node metastasis, patients with metastatic involvement displayed a significantly higher proportion of LAG-3-positive areas (2.906 ± 1.02) compared with non-metastatic patients (1.600 ± 0.89), with the difference reaching statistical significance (P < 0.05, **Figure 4C**). Similarly, analysis of lymphovascular and perineural invasion revealed that tumors exhibiting invasion had markedly higher LAG-3 expression than those without invasion (2.274 ± 1.02 vs. 1.514 ± 0.92, P < 0.05, **Figure 4D**).

Collectively, these results indicate that LAG-3 expression in cervical cancer tissues is closely associated with clinicopathological features, supporting its utility as a mechanistic/biomarker-associated marker of CD8^+^ T-cell dysfunction and tumor aggressiveness, without making direct claims regarding translational application.

### 3.6 Lag-3 deficiency enhances CD8^+^ T cell proliferation and potentiates cytotoxic function against cervical cancer cells

**Figure 5:**
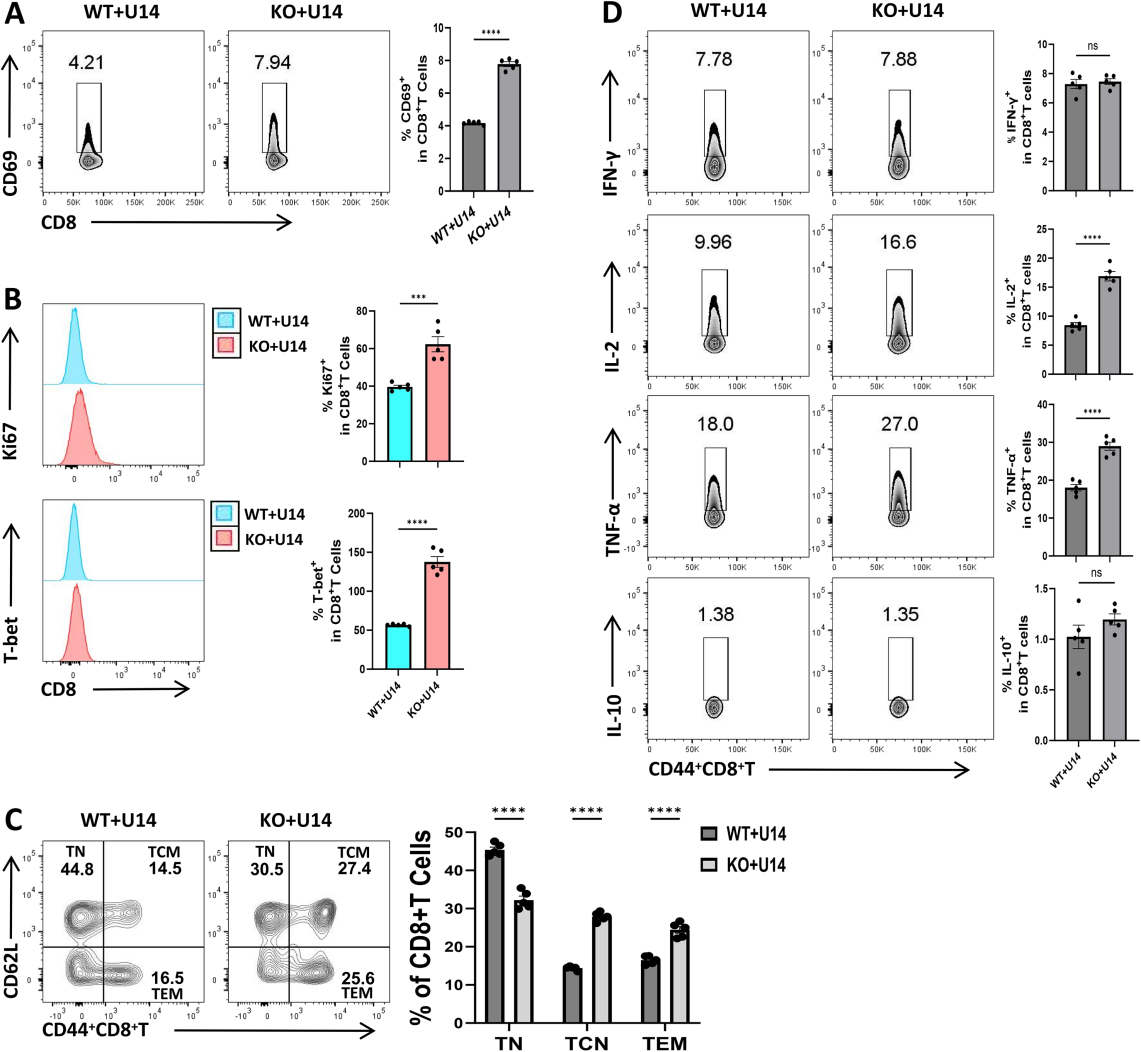
Functional changes in CD8^+^ T cells following co-culture of lag3^+^ and lag-3^-^ CD8^+^ T cells with U14 cervical cancer cells. **(A)** Representative flow cytometry plots and quantification of CD69 expression on CD8^+^ T cells following co-culture.TEM. **(B)** Representative plots and statistical analysis of Ki67 and T-bet expression in CD8^+^ T cells. **(C)** Representative flow cytometry plots and quantification of naive (TN), central memory (TCM), and effector memory CD8^+^ T-cell subsets. **(D)** Representative plots and quantification of TNF-α, IFN-γ, IL-2, and IL-10 secretion by CD44^+^ CD8^+^ T cells. Data are presented as mean ± SEM. WT, lag-3^+^CD8^+^ T cells; KO, lag-3^-^CD8^+^ T cells; Statistical significance: ***P < 0.001, **** P < 0.0001; n.s., not significant (P > 0.05); n = 5 per group.

To investigate the functional impact of lag-3 on CD8^+^ T cells, we generated lag-3^+^ and lag-3^-^ CD8^+^ T cell populations and co-cultured them with cervical cancer U14 cells for 24 hours. Flow cytometry was employed to evaluate T-cell activation, proliferation, and cytokine production following co-culture, thereby delineating the impact of lag-3 deficiency on CD8^+^ T-cell function. Analysis of CD69 expression, a canonical activation marker, revealed that lag3^-^ CD8^+^ T cells displayed significantly higher CD69 levels compared with lag3^+^ CD8^+^ T cells after co-culture (P < 0.05; **Figure 5A**), indicating that lag-3 deficiency enhances T-cell activation.

Analysis of proliferative capacity revealed that lag-3^-^ CD8^+^ T cells exhibited significantly higher Ki67 and T-bet expression following co-culture compared with lag-3^+^ CD8^+^ T cells (P < 0.05; **Figure 5B**), indicating enhanced proliferation and transcriptional activation in the absence of lag-3. Functionally,lag-3^-^ CD8^+^ T cells secreted markedly higher levels of IL-2 and TNF-α relative to lag-3^+^ counterparts (P < 0.05; **Figure 5D**), consistent with previous reports that lag-3 deficiency potentiates CD8^+^ T-cell-mediated anti-tumor immunity (Hirahara et al., 20). No significant differences were observed in IFN-γ or IL-10 production between the two groups (**Figure 5D**).

Evaluation of memory subset distributions demonstrated that lag-3^-^ CD8^+^ T cells exhibited a reduced proportion of naive T cells (TN) and significantly increased frequencies of central memory (TCM) and effector memory (TEM) populations (P < 0.05; **Figure 5C**), indicating that lag-3 deficiency promotes differentiation toward memory subsets with enhanced anti-tumor effector potential. Collectively, these findings demonstrate a mechanistic/biomarker-associated role of lag-3 in constraining CD8^+^ T-cell activation, proliferation, and memory differentiation in response to tumor antigens, emphasizing its relevance as a marker and regulator of T-cell dysfunction rather than a direct therapeutic target (**Supplementary Figures 18**–**28**).

### 3.7 Validation of the regulatory role of lag-3 in CD8^+^ T cell function within the cervical cancer tumor microenvironment in mice

**Figure 6:**
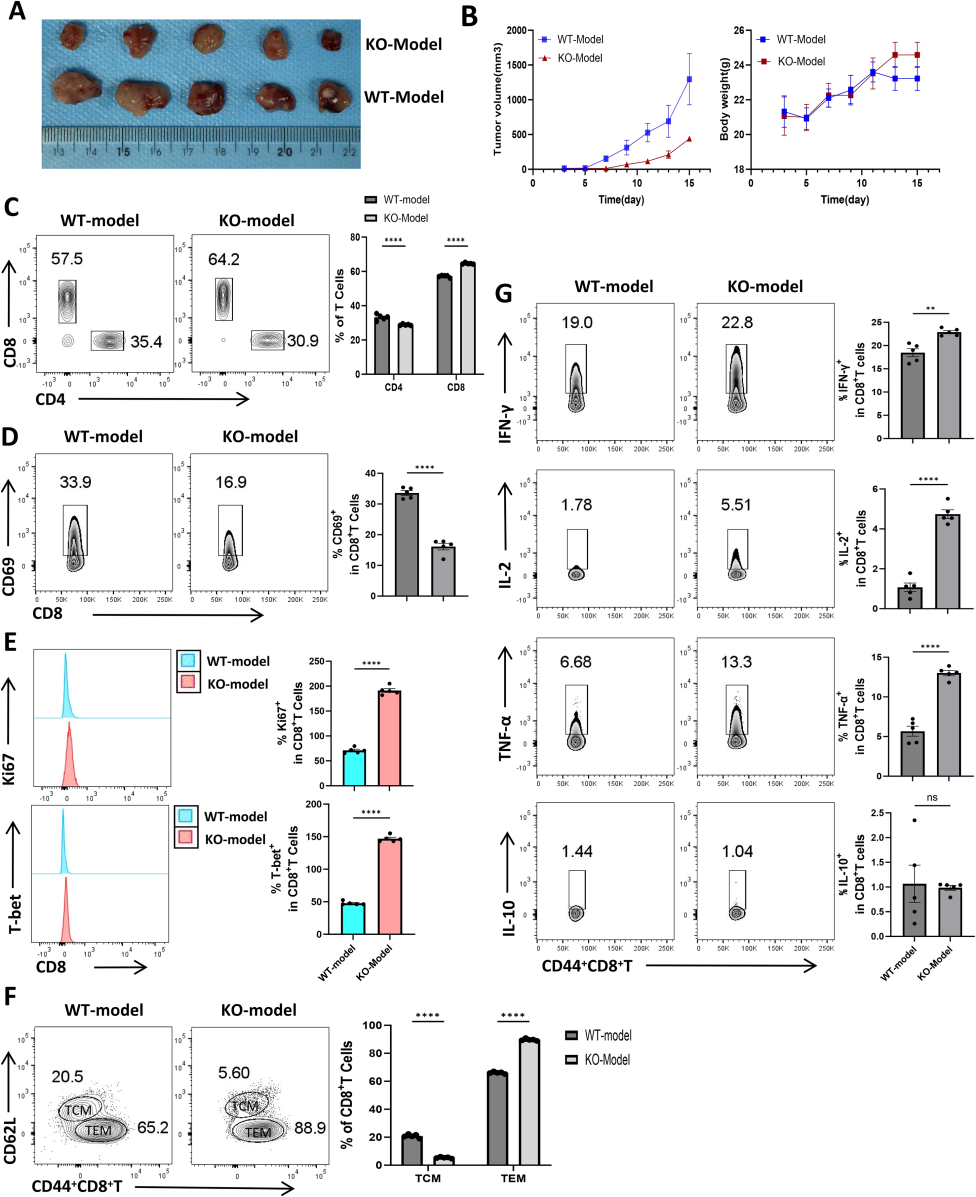
Functional analysis of CD8^+^ T cells in subcutaneous tumors of C57BL/6 wild-type (WT) and lag-3 knockout (KO) mice. **(A)** Representative photographs of subcutaneous tumors from WT and KO mice. **(B)** Tumor growth curves and body weight changes over 15 days for both groups. **(C)** Representative flow cytometry plots and quantitative analysis of CD8^+^ and CD4^+^ T cell proportions within the tumor-infiltrating lymphocyte populations. **(D)** Flow cytometric analysis of CD69 expression in CD8^+^ T cells within the tumor microenvironment, including representative plots and quantification. **(E)** Flow cytometric analysis of Ki67 and T-bet expression in CD8^+^ T cells, showing representative plots and statistical analysis. **(F)** Representative plots and quantification of effector memory (TEM) and central memory (TCM) CD8^+^ T cell subsets in the tumor microenvironment. **(G)** Representative flow cytometry plots and corresponding quantitative analysis of IFN-γ, IL-2, TNF-α, and IL-10 production by CD44^+^ CD8^+^ T cells within the subcutaneous tumor microenvironment of the two mouse cohorts. Each group included five mice aged 8~10 weeks. Data are presented as mean ± standard error of the mean (SEM). WT, C57BL/6 wild-type mice; KO, lag-3-deficient mice. Statistical significance is indicated as: **P < 0.01, **** P < 0.0001; n.s., not significant (P > 0.05).

To systematically evaluate the role of lag-3 in modulating tumor growth and CD8^+^ T cell function *in vivo*, we established subcutaneous cervical cancer syngeneic models in C57BL/6 wild-type (WT) and lag-3-deficient (KO) mice. Tumor growth kinetics revealed that KO mice exhibited markedly delayed tumor progression, with tumor volumes showing significant differences between WT and KO groups by day 15 post-implantation (**Figure 6A**). Body weight monitoring indicated no significant differences between groups (**Figure 6B**), suggesting that lag-3 deficiency suppresses tumor growth without compromising overall health.

Flow cytometric analysis of tumor-infiltrating lymphocytes demonstrated a substantial increase in the proportion of CD8^+^ T cells in KO mice, whereas CD4^+^ T cell infiltration was comparatively reduced (**Figure 6C**). Analysis of CD69, a canonical activation marker on CD8^+^ T cells, revealed that its expression in the tumor microenvironment of lag-3 knockout (KO) mice was significantly reduced compared with wild-type (WT) controls (P < 0.05; **Figure 6D**). This observation contrasts with *in vitro* co-culture results, suggesting that additional regulatory pathways modulate CD8^+^ T-cell activation *in vivo*. Assessment of proliferative capacity via Ki67 and T-bet staining demonstrated enhanced proliferation of CD8^+^ T cells in lag-3^-^/^-^ tumors relative to WT counterparts (**Figure 6E**). Phenotypic analysis further revealed that the proportion of central memory T cells (TCM) among CD8^+^ T cells was decreased in lag-3 KO tumors, whereas effector memory T cells (TEM) were markedly increased (89.7 ± 0.75% versus 66.1 ± 0.66% in WT; **Figure 6F**), indicating that lag-3 restricts the differentiation of CD8^+^ T cells from TCM to TEM.

Functional analysis revealed that CD8^+^ T cells from lag-3^-^/^-^ tumor microenvironments produced significantly higher levels of IFN-γ, IL-2, and TNF-α compared to WT controls (P < 0.05) (**Figure 6G**). Meanwhile, a study by Yuxuan Zhang et al. on esophageal squamous cell carcinoma (ESCC) demonstrated that elevated expression of the immune checkpoint lag-3 is closely associated with T cell immune activation in the ESCC tumor microenvironment, and the increased lag-3 expression inhibits tumor-infiltrating T cells to a certain extent 23.

Collectively, these findings underscore a mechanistic role for lag-3 in modulating CD8^+^ T-cell differentiation and effector function within the cervical cancer tumor microenvironment, emphasizing its utility as a mechanistic/biomarker-associated marker and regulator rather than making direct claims about clinical or translational applications (**Supplementary Figures 18**–**28**).

## 4 Discussion

CD8^+^ T cells are the principal effector lymphocytes mediating anti-tumor immunity and are frequently found to infiltrate a wide spectrum of solid malignancies, highlighting their pivotal role in tumor immune surveillance (28). However, despite their numerical abundance within the tumor microenvironment, tumors often evade immune destruction by establishing a profoundly immunosuppressive milieu that restricts CD8^+^ T cell effector functions, thereby facilitating immune escape (29–31). Immune checkpoint molecules play a central role in this process (32, 33), as they suppress T cell activation, proliferation, and cytokine production through ligand engagement, ultimately driving T cell exhaustion and impairing effective anti-tumor responses (34). In the present study, we focused on the immune checkpoint receptor LAG-3 (lymphocyte-activation gene 3) and its regulatory effects on CD8^+^ T cell function within the cervical cancer TME. Our results demonstrate that CD8^+^ T cells are a dominant population in cervical cancer tissues and exhibit high surface expression of LAG-3, suggesting a critical role of this receptor in modulating T cell anti-tumor activity. Previous studies have established that LAG-3 functions as a negative co-inhibitory receptor on T cells; upon engagement with ligands such as MHC class II or FGL1, it inhibits TCR signaling, thereby constraining T cell activation and effector functions (22, 35). Consistent with our observations, Hirahara et al. reported that CD8^+^ T cells within human cervical cancer exhibit elevated expression of inhibitory immune checkpoint molecules, including LAG-3, particularly within immunosuppressive tumor regions, supporting a conserved role of LAG-3 in shaping dysfunctional T-cell states in the cervical cancer microenvironment (Hirahara et al., British Journal of Cancer) (26). Mechanistically, LAG-3 has been reported to interfere with TCR-mediated signaling cascades at multiple levels. LAG-3 can localize to the immunological synapse in close proximity to the CD3/TCR complex, where it reduces TCR signal strength and sensitivity to antigen stimulation. Although LAG-3 lacks a classical ITIM motif, its unique intracellular KIEELE motif has been implicated in negative regulation of proximal TCR signaling events, including attenuation of ZAP70, LAT, and downstream MAPK/ERK activation. Such suppression of TCR signaling ultimately limits T cell proliferation, cytokine production, and effector differentiation, while promoting the establishment of dysfunctional or exhausted transcriptional programs.

Our *in vitro* co-culture assays and *in vivo* cervical cancer mouse models revealed that lag-3 deficiency altered the infiltration patterns of CD8^+^ T cells and was associated with coordinated changes in phenotypic, proliferative, and functional markers, including CD69 expression, Ki67 and T-bet levels, as well as cytokine production (IFN-γ, IL-2, and TNF-α), reflecting conserved immune checkpoint–mediated regulatory mechanisms in CD8^+^ T cells and highlighting the mechanistic/biomarker-associated role of lag-3 in regulating CD8^+^ T cell functional states rather than directly implying therapeutic potential. Notably, CD69 expression was interpreted with caution in this study, as CD69 is a context-dependent marker that may reflect acute T-cell activation, chronic stimulation or exhaustion, as well as tissue residency depending on the cellular context and accompanying phenotypic features. Therefore, CD69 expression alone was not used as a surrogate indicator of enhanced T-cell activation. Instead, CD69 was evaluated in conjunction with proliferative markers (Ki67), transcriptional regulators (T-bet), and effector cytokine production to provide an integrated assessment of CD8^+^ T-cell functional states.

In light of the established role of LAG-3 in dampening TCR signaling, the enhanced activation and cytokine secretion observed in LAG-3-deficient CD8^+^ T cells in our study may be attributable, at least in part, to the release of TCR signal inhibition. However, we acknowledge that direct assessment of proximal TCR signaling events, such as phosphorylation of CD3ζ, ZAP70, or ERK, was not performed in this study and will be an important direction for future investigation. Accordingly, the divergent trends of CD69 expression observed between *in vitro* co-culture conditions and *in vivo* tumor microenvironments are likely attributable to differences in antigen exposure, stimulation duration, and tissue-contextual cues, rather than contradictory biological effects of lag-3 deficiency. Furthermore, lag-3 deficiency promoted the differentiation of CD8^+^ T cells toward effector memory (TEM) subsets, indicating that lag-3 may influence memory differentiation trajectories and the transition from central to effector memory states in a non–antigen-specific manner, without making claims about clinical benefit and focusing on mechanistic/biomarker-associated correlations. While our data suggest a potential role of lag-3 in modulating the differentiation of central memory T cells (TCM) into effector memory T cells (TEM), further immunophenotyping and functional experiments are required to establish causality. Notably, the magnitude and duration of TCR signaling are critical determinants of CD8^+^ T cell fate decisions. Chronic attenuation of TCR signaling by inhibitory receptors such as lag-3 may restrict effector-memory differentiation, whereas relief of this inhibition could favor the generation and maintenance of TEM populations. The increased TEM differentiation observed upon lag-3 deficiency in our model is therefore consistent with a role for lag-3-mediated TCR suppression in shaping CD8^+^ T cell differentiation trajectories. These findings suggest that lag-3 not only limits initial T-cell activation but also affects memory differentiation and functional states, representing a key regulatory node in CD8^+^ T cell biology. Consistent with previous reports across multiple solid tumors, elevated expression of lag-3 and its ligand FGL1 correlates with immunosuppressive tumor microenvironments. Our observations in the cervical cancer model support the mechanistic relevance of lag-3 in shaping the immune landscape and highlight its potential utility as a mechanistic/biomarker-associated marker of T-cell dysfunction.

Despite the strengths of this study, several limitations should be acknowledged. First, although our conclusions are strongly supported by integrative analyses of human cervical cancer scRNA-seq datasets and well-annotated clinical tissue specimens, the majority of functional validation experiments were performed using murine models and *in vitro* co-culture systems. While these approaches provide robust mechanistic insights, the U14 murine cervical cancer model is not driven by HPV antigens and therefore may not fully recapitulate the immunobiology of HPV-driven cervical cancer, particularly virus-specific T-cell responses. In this context, the observed immune effects are more appropriately interpreted as mechanistic and biomarker-associated immune checkpoint regulation and may not fully reflect HPV antigen–specific CD8^+^ T-cell responses directed against viral oncoproteins such as E6 and E7. Future studies employing etiologically relevant models, including HPV16 E6/E7-driven TC-1 cells or orthotopic tumor models, will be important to further evaluate the antigen-specific relevance of lag-3-mediated immune regulation.

Second, although CD69 expression was included in the phenotypic analysis of tumor-infiltrating CD8^+^ T cells, canonical tissue-resident memory (TRM) markers such as CD103 were not assessed. This limits definitive discrimination between tissue residency, activation, and exhaustion states, and therefore warrants cautious interpretation of CD69-associated phenotypes in the tumor microenvironment.

Third, although the human single-cell transcriptomic data clearly demonstrate elevated LAG-3 expression and an exhausted phenotype in tumor-infiltrating CD8^+^ T cells, direct functional validation of LAG-3-mediated suppression in primary human CD8^+^ T cells was not performed in this study. Therefore, future investigations incorporating ex vivo functional assays using freshly isolated human tumor-infiltrating lymphocytes, as well as humanized or patient-derived models, will be essential to further substantiate the translational relevance of our findings. Collectively, these limitations highlight the need for expanded human-centered functional studies to fully elucidate the role of LAG-3 in regulating CD8^+^ T cell immunity in cervical cancer.

In summary, this study systematically delineates the inhibitory role of LAG-3 on CD8^+^ T-cell functional states in the cervical cancer microenvironment, demonstrating that LAG-3 influences activation, proliferation, cytokine secretion, and differentiation toward the TEM phenotype. These results provide a robust mechanistic foundation and support the consideration of LAG-3 as a mechanistic/biomarker-associated marker for CD8^+^ T cell exhaustion and functional heterogeneity in cervical cancer.

## Data Availability

The original contributions presented in the study are included in the article/**Supplementary Material**. Further inquiries can be directed to the corresponding authors.
