# Supplementary material for: LAG-3–associated CD8+ T-cell dysfunction in the cervical cancer tumor microenvironment
Source: Front Immunol. 2026 Jan 28;17:1750726. doi: 10.3389/fimmu.2026.1750726 (PMC12891123; doi:10.3389/fimmu.2026.1750726)
Supplement: Supplementary file 1 [file DataSheet1.docx]

**Single-cell RNA-seq data processing, integration, and statistical analysis pipeline**

**Pipeline**：

Single-cell RNA sequencing (scRNA-seq) data processing and analysis were performed using (Seurat v4.3.0, SeuratObject v4.1.3, harmony v1.2.0, ggolot2 v3.4.2, patchwork v1.1.3, dplyr v1.1.2, tidyr v1.3.0, GGally v2.1.2, pheatmap v1.0.12, tidyverse v2.0.0, rstatix v0.7.2, ggpubr 0.6.0, ggsignif v0.6.4, future v1.58.0) in R v4.1.3 unless otherwise stated. Raw sequencing data were first subjected to quality control (QC) to remove low-quality cells: cells were retained if they met the following criteria: number of detected genes (features) ≥ 200, number of unique molecular identifiers (UMIs, total RNA counts) ≥ 200, and mitochondrial gene percentage (mt%) ≤ 10%. Additionally, genes expressed in fewer than 3 cells were excluded from the count matrix prior to cell-level QC (filter applied during Seurat object initialization). Batch effects between different patient samples (grouped by the 'orig.ident' metadata field representing individual patients) were corrected using the Harmony algorithm implemented in Seurat with the following parameters: prior to batch correction, the scRNA-seq data were normalized using SCTransform; principal component analysis (PCA) was then performed with 50 principal components (npcs=50, verbose mode disabled), and Harmony batch correction was run with group.by.vars='orig.ident' (to align patient-specific batch effects), assay.use='SCT' (using the SCT assay for correction), and max.iter.harmony=20 (maximum iteration number set to 20). The effectiveness of batch correction was evaluated by visual comparison of UMAP plots generated before and after the Harmony correction step. Dimensionality reduction was performed by PCA on the 3000 highly variable genes (HVGs) identified using Seurat’s FindVariableFeatures function with selection.method="vst", followed by UMAP for visualization using the default parameters of Seurat’s RunUMAP function. Cell clustering was conducted using the Louvain algorithm (default parameters as implemented in Seurat’s FindClusters function) with a resolution parameter of 0.8 (default value in Seurat), resulting in 33 distinct cell clusters. Marker genes for each cluster were identified using Seurat’s FindAllMarkers function with default parameters. Cell types were annotated through two complementary approaches: 1) the expression pattern of known canonical marker genes, where the selection of marker genes was based on reference to the published literature (Xia, 2025) (detailed marker genes for each cell type are listed in Table 1: The marker genes of all cells).

Cell type annotation for NK cells and T cells was performed using module score analysis: canonical marker gene sets for NK cells (NKG7, GNLY, KLRD1, KLRF1, FCGR3A) and T cells (CD3D, CD3E, CD3G, CD4, CD8A) were defined, and module scores for each signature were calculated using Seurat’s AddModuleScore function (default parameters), generating NK_score1 and T_score1 for each cell. Cells were initially annotated as "NK_cells" (if NK_score1 > T_score1) or "T_cells" (if T_score1 > NK_score1). A high-confidence filter was applied using an absolute score difference threshold of 0.3: cells with |NK_score1 - T_score1| > 0.3 retained their annotation (stored in cell_type_conf), while cells with |NK_score1 - T_score1| ≤ 0.3 were labeled as "Low_confidence".

Following global dataset processing and cell type annotation, T cells were subset from the full dataset for fine-grained subclustering analysis. The T cell subset (denoted as t_cells) was processed with the following pipeline: normalization was performed using Seurat’s NormalizeData function (default parameters), followed by identification of 3000 highly variable genes (HVGs) via FindVariableFeatures (nfeatures = 3000). The expression matrix was scaled using ScaleData (default parameters), and dimensionality reduction was conducted via PCA with 30 principal components (npcs = 30, verbose mode disabled). Non-linear dimensionality reduction for visualization was performed using UMAP with custom parameters: dims = 1:30, n.neighbors = 10, and min.dist = 0.8. For subclustering, nearest neighbor graph construction was performed using FindNeighbors with dims = 1:20, and subsequent cell subclustering was conducted via the Louvain algorithm (implemented in Seurat’s FindClusters function) with a resolution parameter of 1.0, generating T cell-specific subclusters for downstream analysis.

Classification and annotation of T cell subclusters were performed using Seurat’s AddModuleScore function (default parameters). The marker gene sets used for score calculation were selected based on reference to the published literature (Wen, 2024), with detailed marker genes for each T cell subtype listed in Table 3: The marker genes of T cells.

To assess inter-sample differences in gene expression levels across T cell subtypes, the pairwise Wilcoxon rank-sum test was implemented in the pairwise_wilcox_test function. Multiple testing corrections were applied to P-values using the Benjamini-Hochberg (BH) method to control the false discovery rate (FDR). For visualization of differential expression results, only the comparison groups with adjusted P-values (p.adj) < 0.05 were annotated in the final plots.

Correlation analysis of gene expression levels and state scores was performed by calculating a Pearson correlation matrix using R’s cor function. Missing values were handled by retaining only complete observations (parameter: use = "complete.obs").

### Differential analysis of patient-level aggregated gene expression across T cell subtypes was conducted via a custom function (cell_type_test): for each T cell subtype, the dataset was filtered to retain disease groups with ≥1 patient, and statistical testing was only performed if ≥2 disease groups were available. A Kruskal-Wallis test assessed global expression differences across sample groups, and non-redundant pairwise comparisons were performed using the Mann-Whitney U test (exact = FALSE). Raw P-values from pairwise tests were adjusted via the Benjamini-Hochberg (BH) method, and results were annotated based on adjusted P-values (significant if p.adj < 0.05). For <2 disease groups, default notes were assigned to indicate insufficient data.

**References:**

[1] Xia, P., Zhou, J., Shen, R. et al. Deciphering the cellular and molecular landscape of cervical cancer progression through single-cell and spatial transcriptomics. npj Precis. Onc. 9, 158 (2025).

[2] Wen Z, Wang L, Ma H, Li L, Wan L, Shi L, Li H, Chen H, Hao W, Song S, Xue Q, Wei Y, Li F, Xu J, Zhang S, Wong KW, Song Y. Integrated single-cell transcriptome and T cell receptor profiling reveals defects of T cell exhaustion in pulmonary tuberculosis. J Infect. 2024 Jun;88(6):106158.

### ****1.Data source and quality control****

Single-cell RNA-sequencing data were obtained from the publicly available dataset **E-MTAB-12305** , comprising a total of **10 samples**, including **normal cervical epithelium** (N, n = 3), **high-grade squamous intraepithelial lesions (HSIL)** (H, n = 2), **primary cervical tumors** (T, n = 4), and **metastatic lymph node tissue** (L, n = 1).

Prior to quality control, sample **H1** contained 23,512 genes and 6,947 cells; **H2**, 24,976 genes and 13,250 cells; **L1**, 23,163 genes and 10,043 cells; **N1**, 24,742 genes and 14,755 cells; **N2**, 24,664 genes and 13,926 cells; **N3**, 22,106 genes and 11,445 cells; **T1**, 23,930 genes and 11,818 cells; **T2**, 24,459 genes and 8,694 cells; **T3**, 21,190 genes and 13,247 cells; and **T4**, 24,122 genes and 10,433 cells.

After integration of all samples, the combined dataset comprised **114,558 cells and 28,784 genes**. The distributions of the number of detected genes (nFeature), total UMI counts (nCount), mitochondrial gene fraction (percent.mt), and ribosomal gene fraction (percent.rb) prior to quality control are shown in ****Figure 1****.


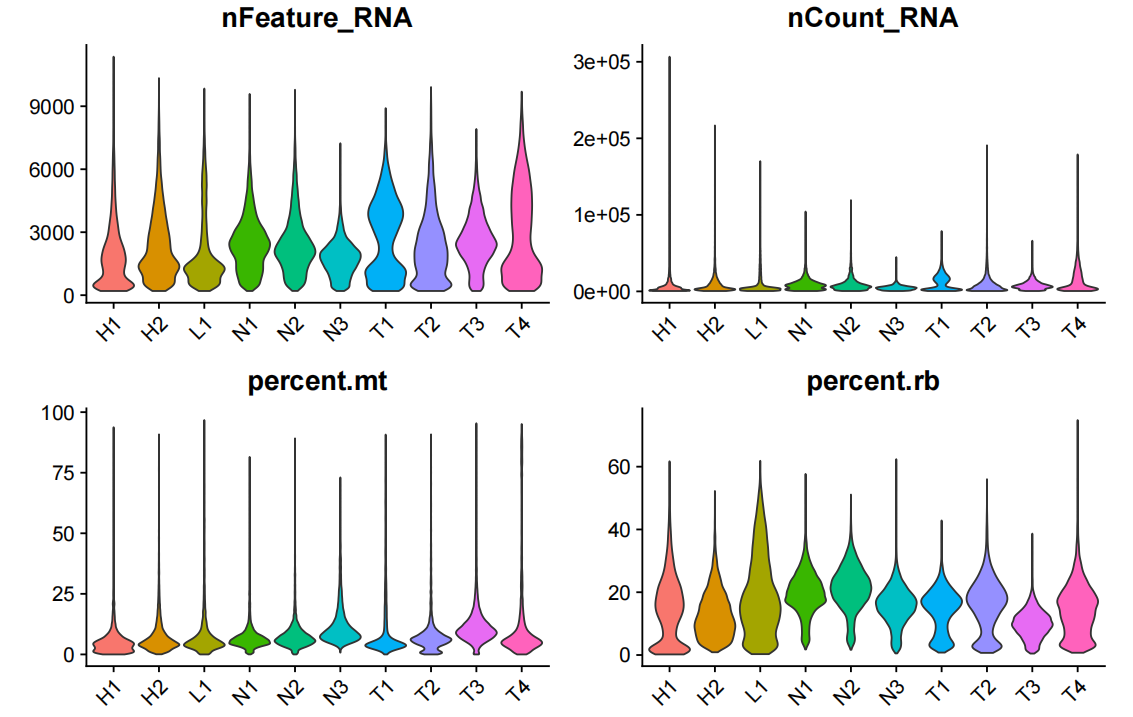
Figure 1: nFeatures, nCount, percent.mt and percent.rb before Quality Control

Quality control was performed according to previously published criteria . Specifically, cells with fewer than 200 detected UMIs or with a mitochondrial gene fraction greater than 10% were excluded from downstream analyses. After quality filtering, 28,784 genes and 83,129 cells were retained (Figure 2). The remaining cells exhibited high-quality transcriptomic profiles and were therefore considered suitable for subsequent analyses.


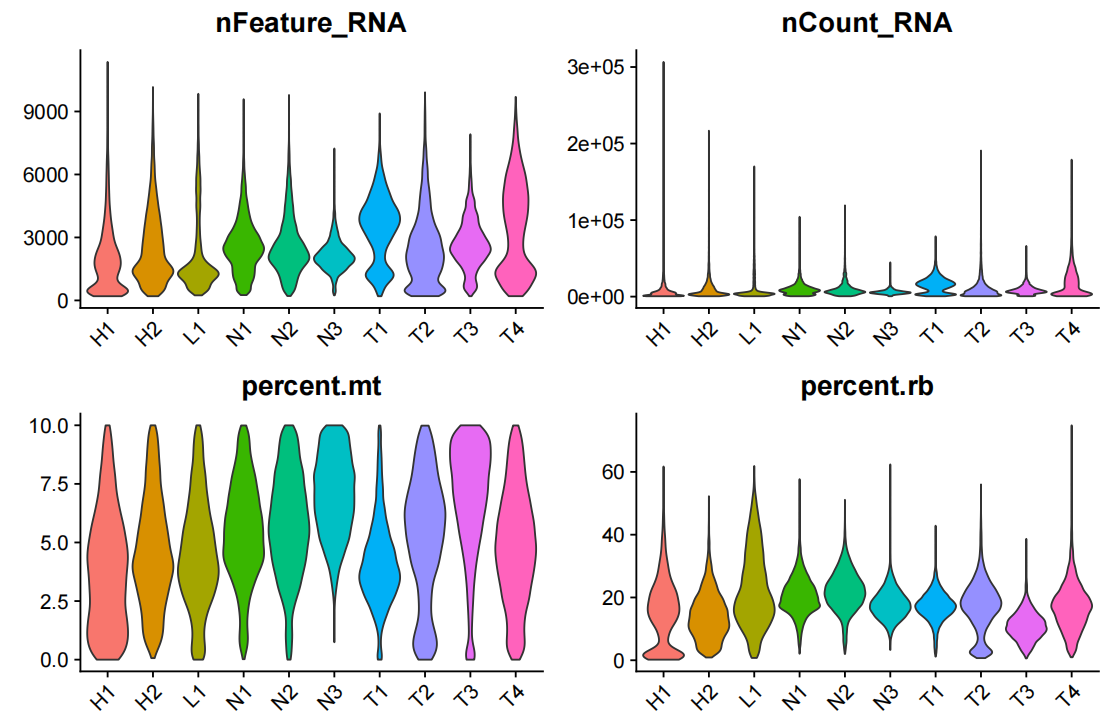
Figure 2: nFeatures, nCount, percent.mt and percent.rb before Quality Control

Data normalization and scaling were performed in accordance with the standard Seurat workflow , with the aim of mitigating technical variation, harmonizing expression scales across cells, and improving the robustness of downstream analyses. This preprocessing ensured high-quality input data for subsequent biological inference.

The Elbow plot was used to guide the selection of the dimensionality for principal component analysis (PCA). As the number of principal components increases, the corresponding variance explained typically decreases, with a marked inflection point indicating diminishing returns. Based on this criterion, 30 principal components were selected as the optimal balance between information retention and model complexity for downstream dimensionality reduction and clustering (Figure 3).


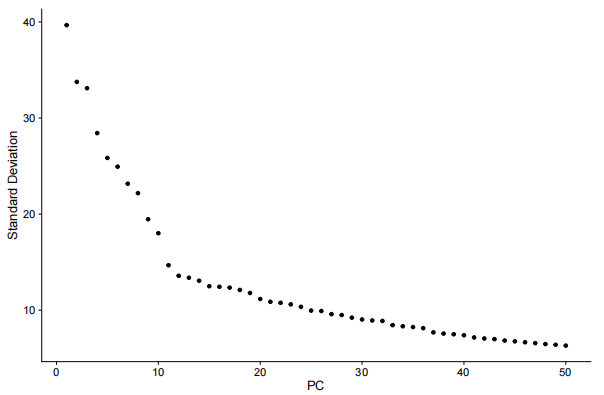


Figure 3: Elbow plot of all samples

The Harmony algorithm was applied to integrate the scRNA-seq datasets, thereby correcting batch effects while preserving genuine biological variation across cells. Prior to integration, cells from individual samples were relatively compact within samples but displayed clear separation across different samples (Figure 4). Consistently, visualization of the four sample types before integration revealed pronounced batch-associated differences (Figure 5). Following Harmony-based integration, cells from different samples and sample types were well aligned in the low-dimensional space, indicating effective correction of batch effects

(Figs. 6 and 7 ).


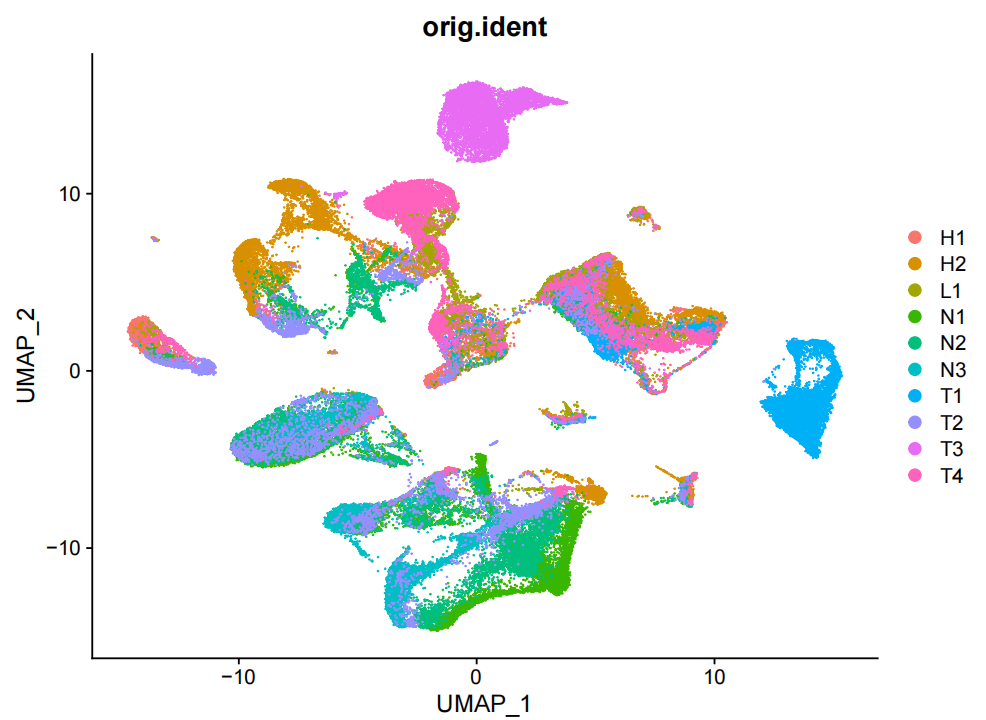


Figure 4: The dimensionality reduction result of all samples before Harmony


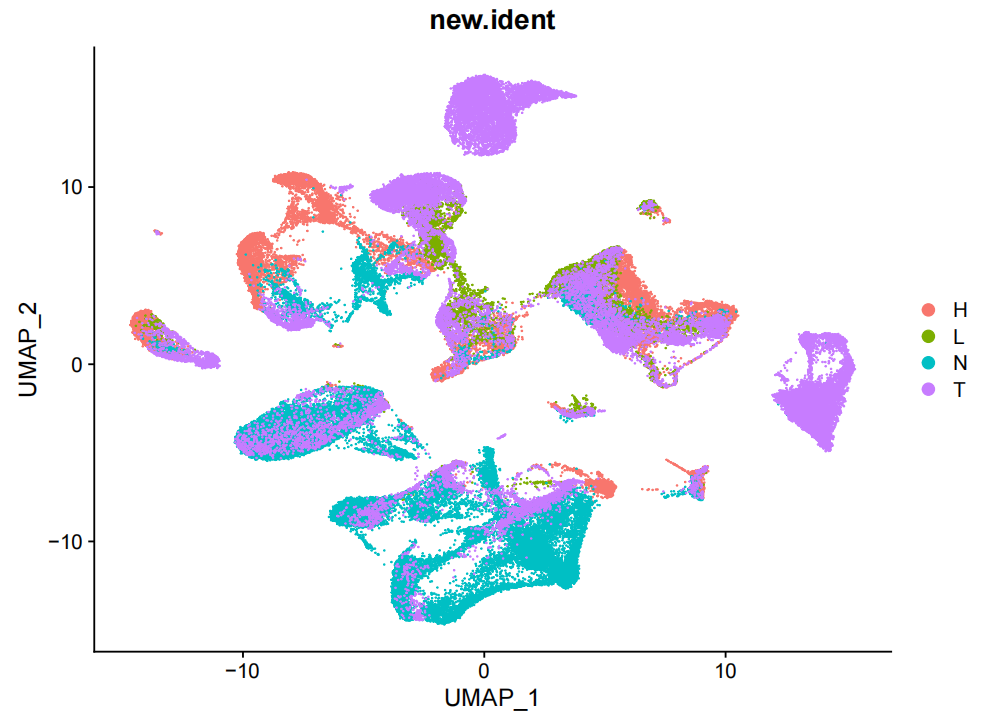


Figure 5: The dimensionality reduction result of sample types before Harmony


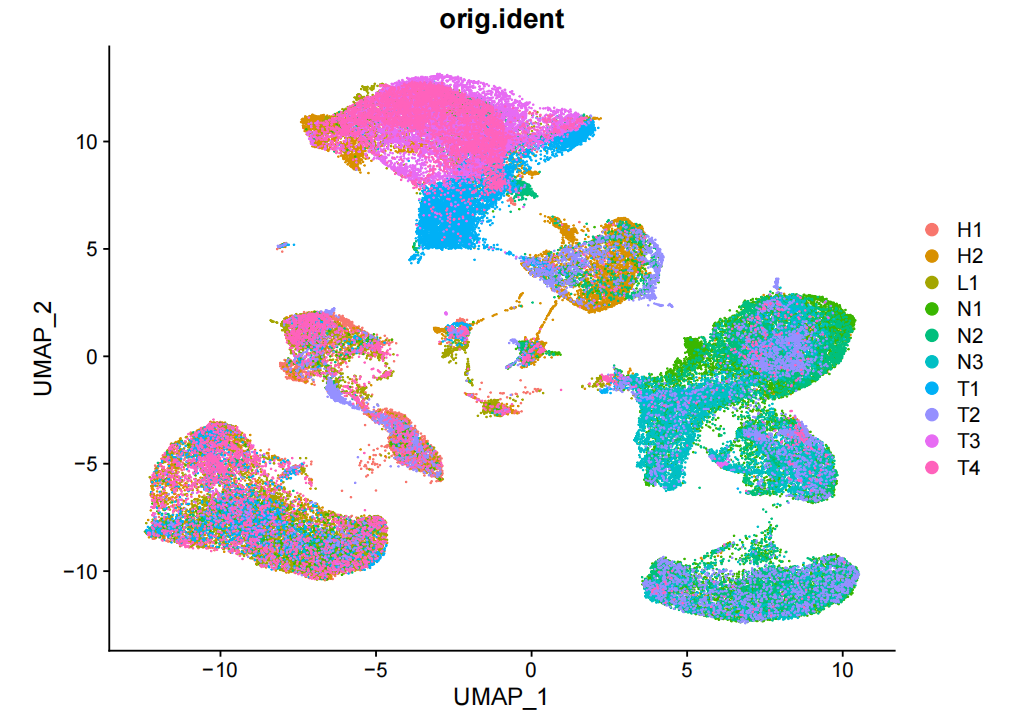


Figure 6: The dimensionality reduction result of all samples after Harmony


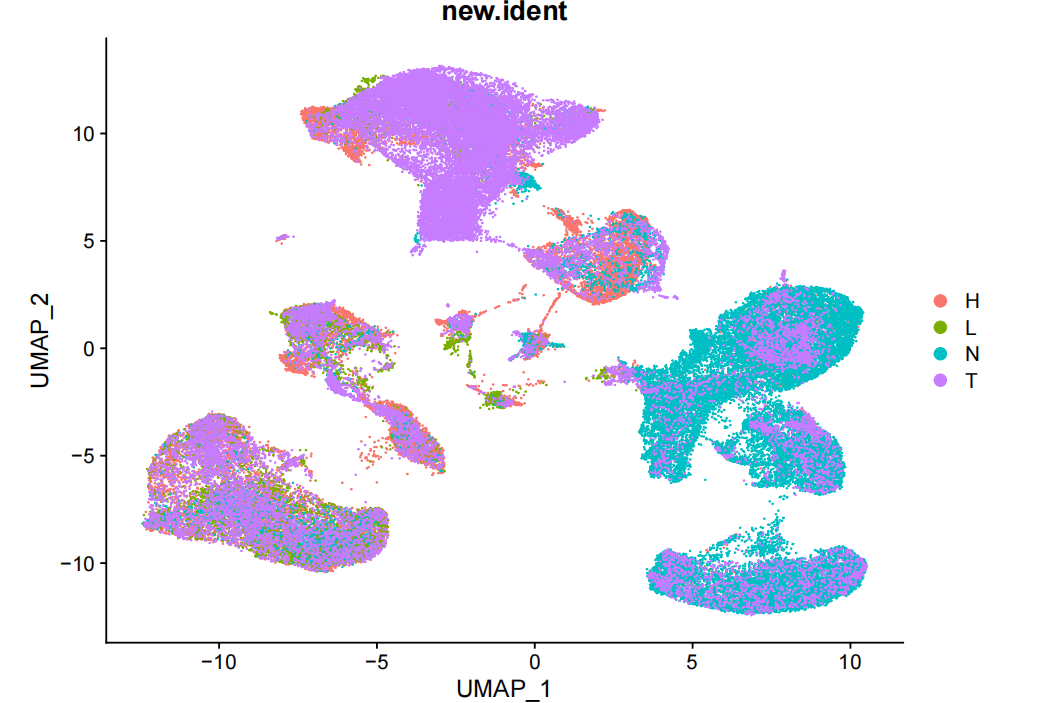


Figure 7: The dimensionality reduction result of sample types after Harmony

###

### ****2.Clustering and subpopulation identification****

Uniform Manifold Approximation and Projection (UMAP) was used to visualize the clustering results of the cervical cancer samples (Figure 8). In total, **33 distinct cell clusters** were identified. Subsequently, **three rounds of stepwise subclustering** were performed to further delineate cellular subpopulations for downstream analyses.


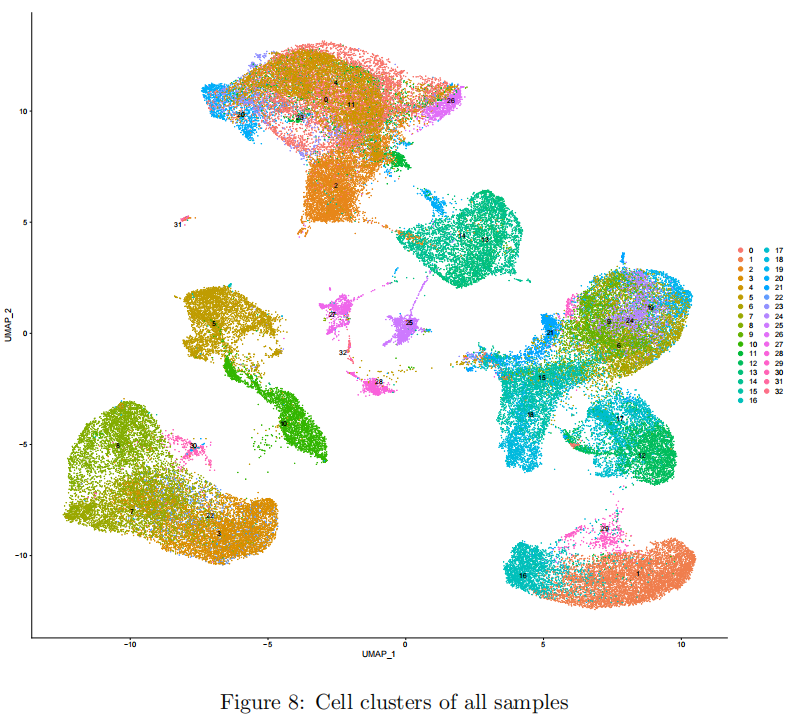


Figure 8: Cell clusters of all samples

### ****2.1 Identification of major cell types and extraction of NK/T cells****

The first round of subclustering focused on annotation of major cell lineages. Based on established markers reported in previous studies , all cells were classified into **10 major cell types**, including **epithelial cells, fibroblasts, endothelial cells, smooth muscle cells, myeloid cells, NK/T cells, neutrophils, B cells, mast cells, and plasma cells**. The marker genes defining each cell type are summarized in **Table 1**.

Expression patterns of these canonical marker genes were visualized to facilitate cell type identification. **Figure 9** depicts the proportion and intensity of marker gene expression across individual clusters, enabling robust discrimination of known cell types.

Following annotation, the low-dimensional distribution of each cell type is shown in **Figure 10**. Consistent spatial concordance was observed between the localization of annotated cell populations and the high-expression regions of their corresponding marker genes. **Figure 11** further illustrates the expression profiles of marker genes across annotated cell types, highlighting distinct lineage-specific expression patterns and supporting the reliability of the annotation strategy. Finally, the relative proportions of each major cell type across the four sample groups are summarized in **Figure 12**.

Table 1: The marker genes of all cells

| **Cluster** | **Genes** |
| --- | --- |
| Epithelial cells | EPCAM, KLF5, MKI67 |
| Fibroblasts | DCN, COL1A1, COL3A1 |
| Endothelial cells | PECAM1, CDH5, VWF |
| Smooth muscle cells | ACTA2, RGS5 |
| Myeloid cells | CD68, CSF1R, CD163, LYZ |
| NK/T cells | NKG7, CCL5, GZMA, CD3G, CD3E, CD3D |
| Neutrophils | NCF1, SORL1 |
| B cells | CD19, BANK1, MS4A1 |
| Mast cells | TPSAB1, CPA3 |
| Plasma cells | MZB1, IGHG1, IGKC, IGHG3, XBP1, JCHAIN |


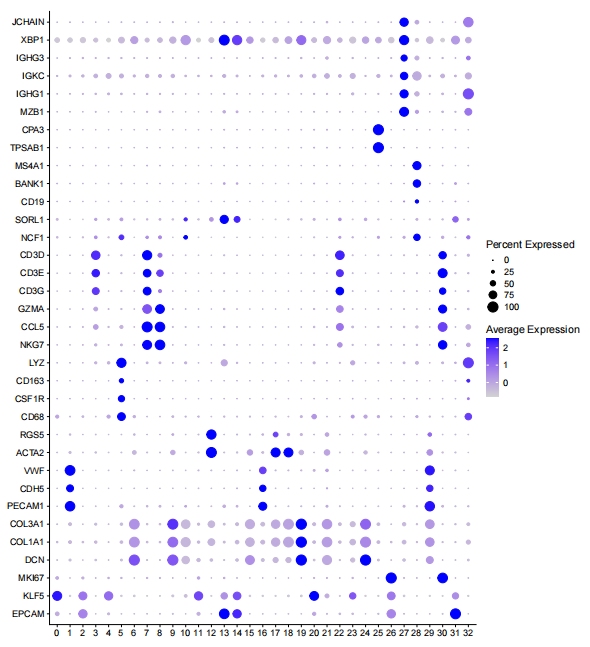


Figure 9: The dimensionality reduction result of all samples


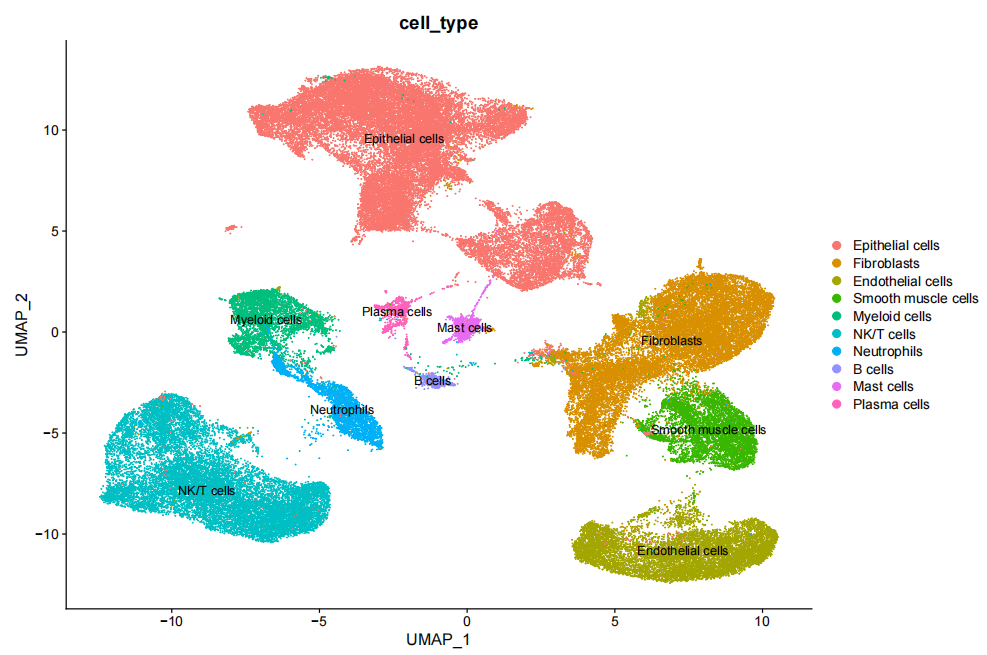


Figure 10: Cell annotation


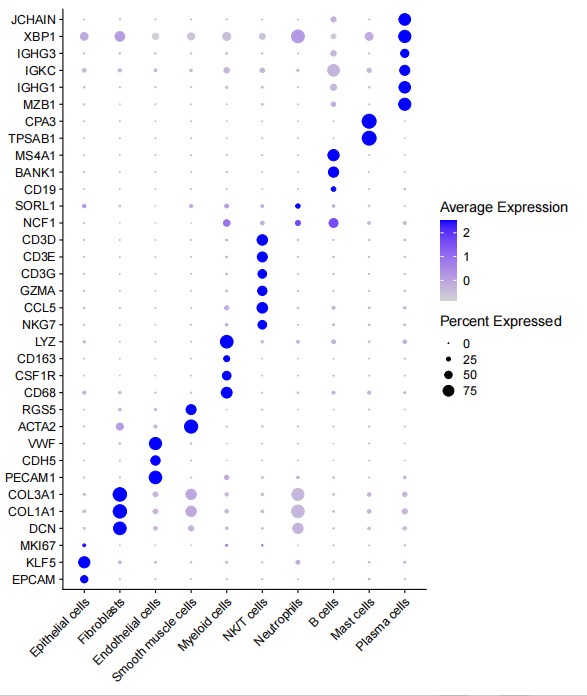


Figure 11: Cell annotation and gene markers


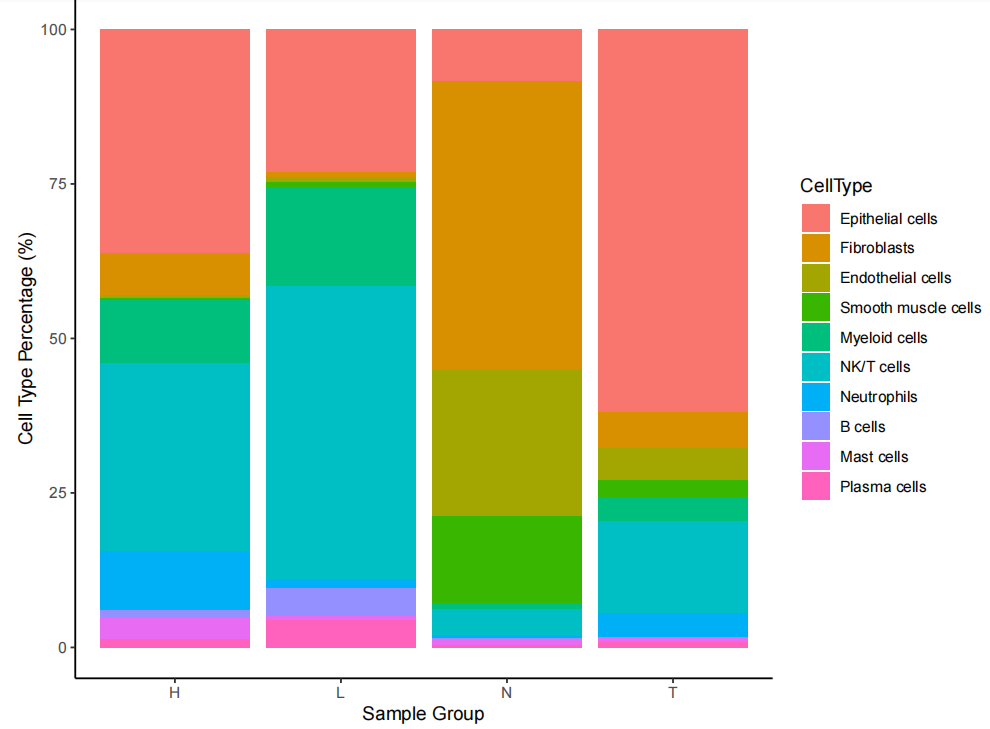


Figure 12: Cell type proportions

**2.2 Extraction and annotation of T cells**

NK/T cells identified in the previous step were further analyzed to distinguish NK cells from T cells. Based on the core marker genes for NK and T cells listed in Table 2, module score analysis was performed using AddModuleScore in Seurat to quantify the activity of lineage-specific gene signatures at the single-cell level. Cell identities were subsequently assigned according to their respective module scores.

The expression patterns of canonical marker genes in the annotated NK and T cell populations are shown in Figure 13, illustrating clear and consistent lineage-specific expression profiles and supporting the accuracy of the NK/T cell classification.

Table 2: The marker genes of NK/T cells

| **Cluster** | **Genes** |
| --- | --- |
| NK cells | NKG7, GNLY, KLRD1, KLRF1, FCGR3A |
| T cells | CD3D, CD3E, CD3G, CD4, CD8A |


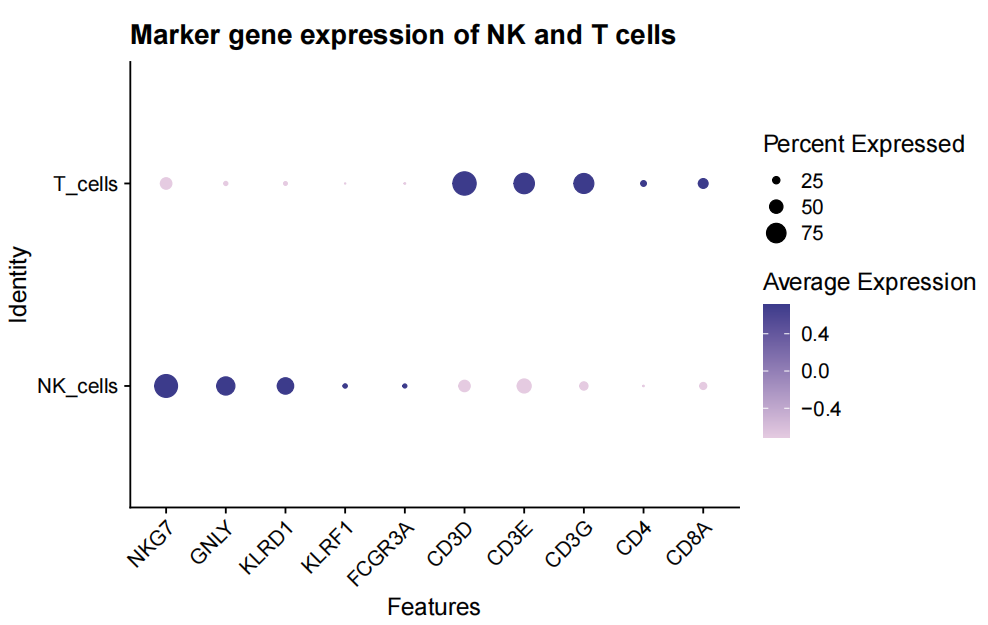


Figure 13: Cell annotation and gene markers of NK/T cells

### **2.3 Extraction of CD8⁺ T cells**

Following the extraction of T cells, T cell subsets were further annotated based on established marker genes reported in previous studies. The marker genes defining each T cell subset are summarized in Table 3. Module score analysis was performed using AddModuleScore in Seurat to quantify the activity of subset-specific gene signatures at the single-cell level, and cell identities were subsequently assigned according to their respective scores.

The low-dimensional distribution of the annotated T cell subsets is shown in Figure 14, illustrating clear separation and structured organization of distinct T cell populations within the T cell compartment.

Table 3: The marker genes of T cells

| **Cluster** | **Genes** |
| --- | --- |
| CD4 Naive | CCR7, SELL, TCF7, IL7R, GPR183, LEF1, LTB |
| CD4 Treg | FOXP3, IL2RA, CTLA4, TIGIT, TNFRSF18, MAGEH1, SAT1, CCR8, IL10, BATF |
| CD4 Th1 | UCP2, TBX21, IFNG |
| CD4 Th17 | ADAM19, RORA, IL17A, IL17F, RORC, IL23R, CTSH |
| CD4 Tfh | CXCL13, CTLA4, PDCD1, ICOS, TNFRSF8, BCL6, TOX |
| CD4 Memory | CCR7, HSPA6, MT1E, MT1F, CD69, GPR183, IL7R, KLF2, TOB1, RPS19, DUSP2, CD44 |
| CD8 Naive | CCR7, SELL, S100A8, CST3, IL7R, LEF1, TCF7 |
| CD8 Central Memory | RPS26, GZMK, CD44, EOMES, CD28, CCR7, DKK3 |
| CD8 Tex | CXCL13, TIGIT, CTLA4, PDCD1, LAG3, HAVCR2, ENTPD1, TOX, TOX2, LAYN, TNFRSF9, TCF7 |
| CD8 Trm | XCL1, XCL2, IL7R, PRDM1, TGFBR2, ITGAL, CCR7, SELL |
| CD8 Effector | DUSP2, GZMK, CX3CR1, NKG7, PRF1, GZMK, GZMA, GZMB, GZMH, IFNG, FGFBP2, FCGR3A, GNLY |
| CD8 StressResponse | FGFBP2, ADGRG1, FCGR3A, HSPA1A, HSPA1B, CD27 |
| CD8 pTex | TCF7, CD27, CD28, EOMES |
| MAIT | TRAV1-2, SLC4A10, GZMK, KLRB1, RORC, RORA |
| NKT | EOMES, XCL1, XCL2, CXCR6, TIGIT, LAG3 |
| IEL/DNT | CD160, KIR2DL4, TMIGD2, ITGAE, GZMK |


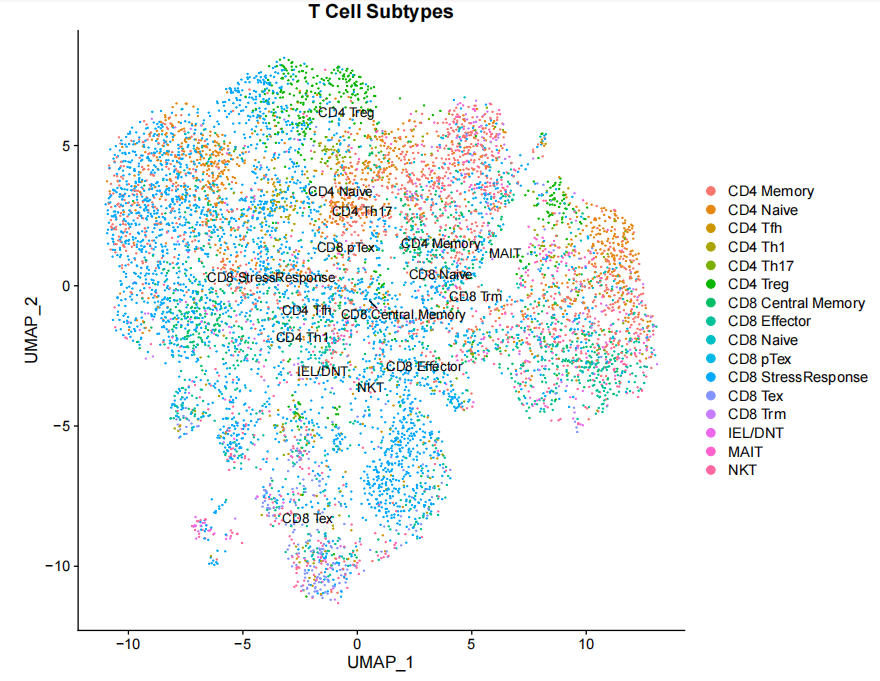


Figure 14: Cell cluster of T cells

### **3.Analysis of CD8⁺ T cells**

Based on the T cell subset annotation described above, the relative proportions of CD8⁺ T cell subsets are summarized in Figure 15. In HSIL samples (H), CD8 effector and CD8 stress-response populations were enriched. In contrast, CD8 stress-response cells represented the predominant subset in metastatic lymph node samples (L). Compared with these disease-associated samples, normal cervical tissues (N) exhibited a higher proportion of CD8 central memory and CD8 tissue-resident memory (Trm) cells.

These distribution patterns are consistent with the known functional characteristics of the corresponding subsets, as defined by their marker gene expression profiles (Table 3), and collectively suggest progressive remodeling of the CD8⁺ T cell compartment across different disease states.


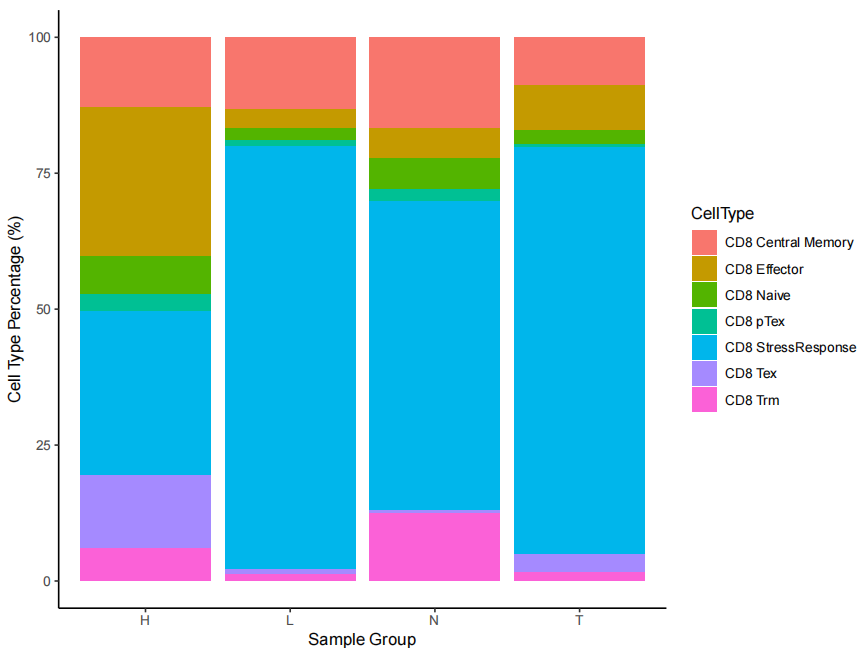


Figure 15: Cell proportion of CD8+T cells

We initially performed group-wise comparisons at the single-cell level and observed statistically significant differences in LAG3 expression among disease categories. However, because cells derived from the same patient are not independent biological replicates, such analyses are susceptible to pseudo-replication. We therefore re-evaluated LAG3 expression after aggregating data at the patient level and re-performing statistical tests.

Under patient-level aggregation, no statistically significant differences in LAG3 expression were detected across disease groups. This discrepancy highlights the impact of pseudo-replication on statistical inference: the apparent significance observed at the cell level likely reflects inflation of sample size due to non-independent observations rather than true biological differences between disease states. By contrast, patient-level analysis, which represents the gold standard for inference across clinical groups, indicates that LAG3 expression does not reach the threshold of systematic, cross-patient differences among disease categories.

It should be noted that the number of patients per disease group in this study was limited (≤4 per group), resulting in constrained statistical power and an increased risk of type II error. Consequently, while patient-level analysis suggests the absence of robust disease-associated differences in LAG3 expression, we cannot fully exclude the possibility of small effect-size differences between disease groups. Future studies with larger patient cohorts will be required to definitively assess the association between LAG3 expression and disease status.


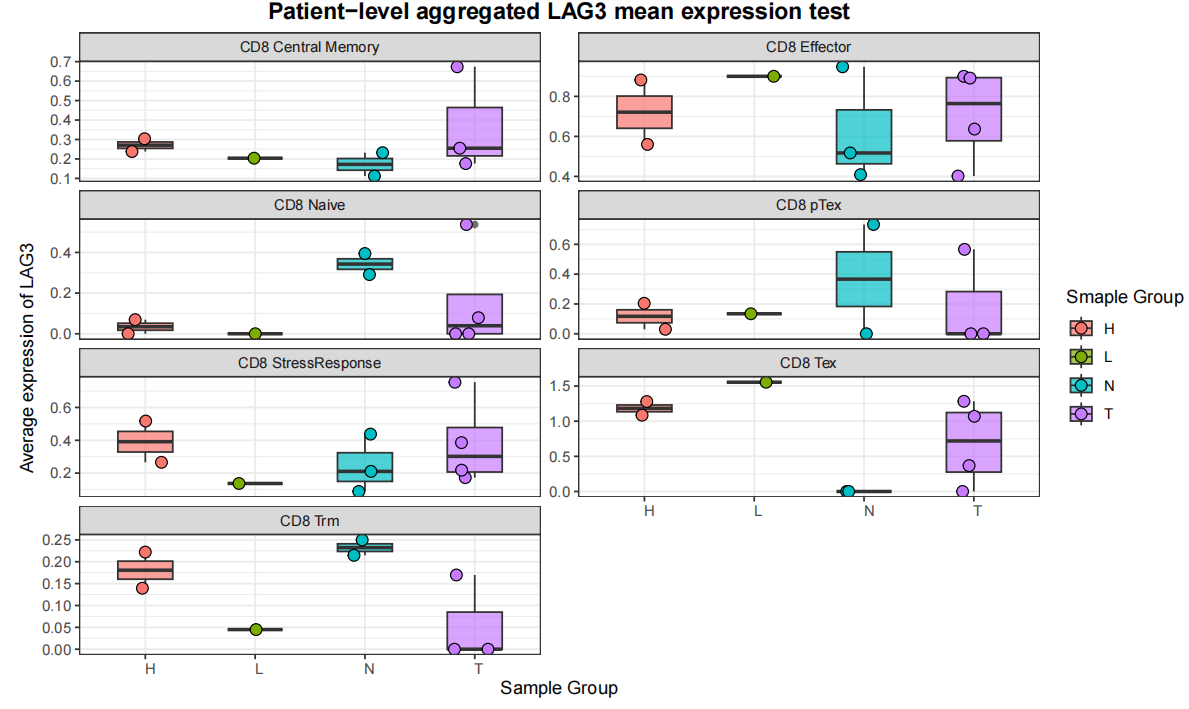
Figure 16: Patient-level aggragated test of lag3 average expression


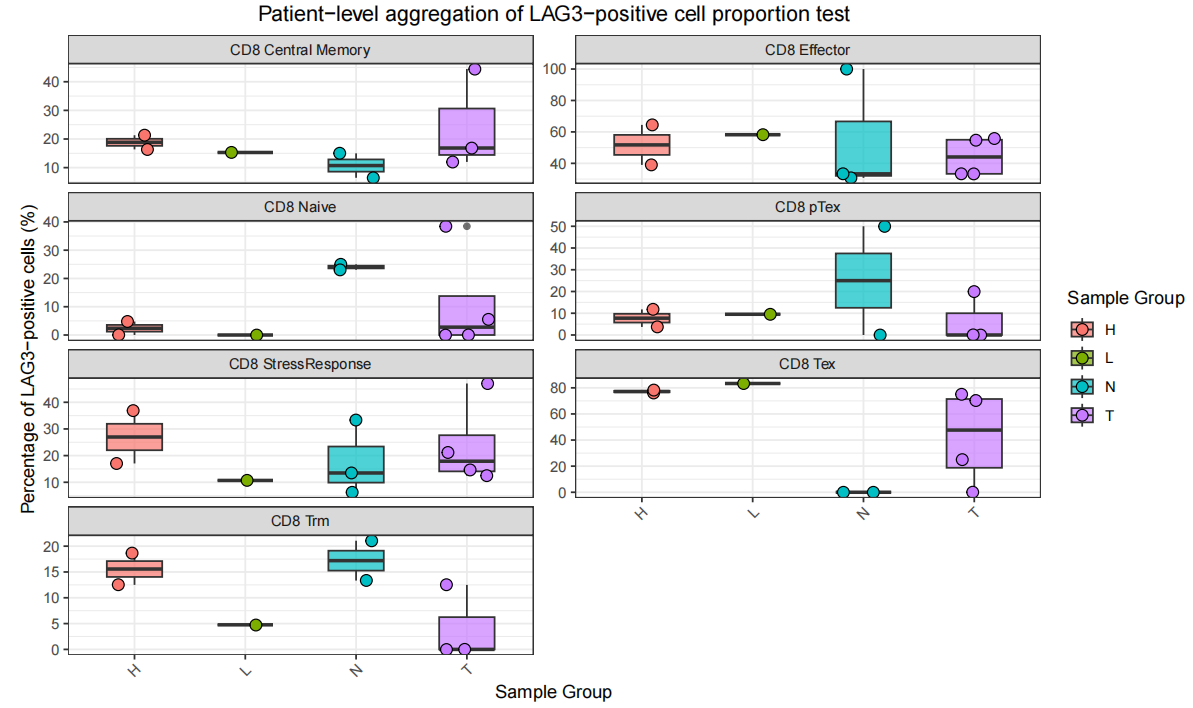
Figure 17: Patient-level aggragated test of lag3 positive cell proportions

**Code R：**

Reproducible scRNA-seq analysis pipeline with sample-level statistics（）

############################################################

## scRNA-seq analysis pipeline (Seurat)

## Purpose:

## 1) Fully reproducible workflow

## 2) Explicit QC / filtering / clustering parameters

## 3) Sample-level aggregation to avoid pseudo-replication

############################################################

## -----------------------------

## 0. Environment

## -----------------------------

set.seed(1234)

library(Seurat)

library(harmony)

library(dplyr)

library(ggplot2)

library(patchwork)

## -----------------------------

## 1. Input data

## -----------------------------

## Assume one Seurat object containing all cells

## Metadata must include:

## sample_id : unique patient/sample identifier

## group : clinical group (e.g. Normal / HSIL / LN)

sce <- readRDS("scRNA_all_samples.rds")

## -----------------------------

## 2. Quality control (QC)

## -----------------------------

## Mitochondrial genes

sce[["percent.mt"]] <- PercentageFeatureSet(

sce, pattern = "^MT-"

)

## QC thresholds (defined a priori)

## - nFeature_RNA: 200–6000

## - nCount_RNA : < 50,000

## - percent.mt : < 15%

sce <- subset(

sce,

subset =

nFeature_RNA > 200 &

nFeature_RNA < 6000 &

nCount_RNA < 50000 &

percent.mt < 15

)

## -----------------------------

## 3. Normalization & HVGs

## -----------------------------

sce <- NormalizeData(

sce,

normalization.method = "LogNormalize",

scale.factor = 10000

)

sce <- FindVariableFeatures(

sce,

selection.method = "vst",

nfeatures = 2000

)

## -----------------------------

## 4. Scaling & PCA

## -----------------------------

sce <- ScaleData(

sce,

vars.to.regress = c("nCount_RNA", "percent.mt")

)

sce <- RunPCA(

sce,

features = VariableFeatures(sce),

npcs = 50

)

## -----------------------------

## 5. Batch correction

## -----------------------------

## Batch variable: sample_id

sce <- RunHarmony(

sce,

group.by.vars = "sample_id",

reduction = "pca"

)

## -----------------------------

## 6. Clustering & UMAP

## -----------------------------

sce <- FindNeighbors(

sce,

reduction = "harmony",

dims = 1:30

)

## Clustering resolution explicitly reported

sce <- FindClusters(

sce,

resolution = 0.5

)

sce <- RunUMAP(

sce,

reduction = "harmony",

dims = 1:30

)

## -----------------------------

## 7. Marker identification

## -----------------------------

## Marker selection rules:

## - Wilcoxon rank-sum test

## - min.pct ≥ 0.25

## - logFC ≥ 0.25

markers <- FindAllMarkers(

sce,

only.pos = TRUE,

min.pct = 0.25,

logfc.threshold = 0.25,

test.use = "wilcox"

)

write.csv(markers, "cluster_markers.csv", row.names = FALSE)

## -----------------------------

## 8. Cell state annotation

## -----------------------------

## Annotation based on canonical marker genes

## (example shown; adapt to your marker list)

cluster.annotation <- c(

"Naive_T",

"CD8_Effector",

"CD8_Exhausted",

"CD8_StressResponse",

"Memory_T",

"Cycling_T"

)

names(cluster.annotation) <- levels(sce)

sce <- RenameIdents(

sce,

cluster.annotation

)

sce$cell_state <- Idents(sce)

## -----------------------------

## 9. Visualization

## -----------------------------

p1 <- DimPlot(

sce,

reduction = "umap",

group.by = "cell_state",

label = TRUE

)

p2 <- DimPlot(

sce,

reduction = "umap",

group.by = "group"

)

p1 + p2

## -----------------------------

## 10. Sample-level aggregation

## -----------------------------

## Avoid pseudo-replication:

## Aggregate cell proportions per sample

cell.prop <- sce@meta.data %>%

group_by(sample_id, group, cell_state) %>%

summarise(n = n(), .groups = "drop") %>%

group_by(sample_id) %>%

mutate(prop = n / sum(n))

write.csv(

cell.prop,

"cell_state_proportions_per_sample.csv",

row.names = FALSE

)

## -----------------------------

## 11. Statistical testing

## -----------------------------

## Example: compare CD8_Effector proportion between groups

effector.prop <- cell.prop %>%

filter(cell_state == "CD8_Effector")

## Non-parametric test at sample level

stat.test <- wilcox.test(

prop ~ group,

data = effector.prop

)

print(stat.test)

## -----------------------------

## 12. Boxplot (sample-level)

## -----------------------------

ggplot(effector.prop, aes(x = group, y = prop)) +

geom_boxplot(outlier.shape = NA) +

geom_jitter(width = 0.2, size = 2) +

theme_classic() +

ylab("Proportion per sample") +

xlab("Group")

### ****Sample-level Kruskal–Wallis analysis of CD8⁺ T-cell subtype proportions****

Supplementary Table 4 summarizes the results of sample-level Kruskal–Wallis tests assessing differences in the proportions of CD8⁺ T-cell subtypes across clinical groups (Normal, HSIL, primary tumor, and metastatic lymph node). For each CD8⁺ T-cell subtype, proportions were first aggregated at the patient/sample level, and group-level differences were then evaluated using non-parametric Kruskal–Wallis tests to avoid pseudo-replication. No statistically significant differences were detected for any CD8⁺ T-cell subtype at the sample level, indicating that while inter-group trends may be observed at the cellular level, these differences do not reach statistical significance when appropriately accounting for biological replication.

Table 4 kruskal wallis result

| t_subtype | kruskal_stat | kruskal_p | kruskal_note |
| --- | --- | --- | --- |
| CD8 Central Memory | 2.80555555555556 | 0.422586190063826 | Not significant |
| CD8 Effector | 1.5 | 0.682270330336213 | Not significant |
| CD8 Naive | 3.18181818181818 | 0.364432921828538 | Not significant |
| CD8 StressResponse | 2.9 | 0.407301567035948 | Not significant |
| CD8 Tex | 5.34482758620689 | 0.14821990234401 | Not significant |
| CD8 Trm | 4.52610441767068 | 0.209973618377558 | Not significant |
| CD8 pTex | 0.583333333333335 | 0.900236999604507 | Not significant |

### ****Sample-level post hoc pairwise comparisons of CD8⁺ T-cell subtype proportions****

Supplementary Table 5 presents post hoc pairwise comparisons between clinical groups for each CD8⁺ T-cell subtype based on sample-level aggregated proportions. Pairwise comparisons were performed using Wilcoxon rank-sum tests, followed by multiple-testing correction to control for false discovery. Consistent with the Kruskal–Wallis results, none of the pairwise comparisons reached statistical significance after adjustment, further supporting that differences in CD8⁺ T-cell subtype distributions are not statistically robust at the patient/sample level. These results underscore the importance of sample-level statistical analysis in single-cell studies and support the conservative interpretation of group-level differences presented in the main text.

Table 5 pairwise comparison result

| CD8 Central Memory | H vs L | 0.54029137460742 | 1 | Not significant |  |
| --- | --- | --- | --- | --- | --- |
| CD8 Central Memory | H vs N | 0.245278116806773 | 1 | Not significant |  |
| CD8 Central Memory | L vs N | 1 | 1 | Not significant |  |
| CD8 Central Memory | H vs T | 1 | 1 | Not significant |  |
| CD8 Central Memory | L vs T | 1 | 1 | Not significant |  |
| CD8 Central Memory | N vs T | 0.386476230771233 | 1 | Not significant |  |
| CD8 Effector | H vs L | 0.54029137460742 | 1 | Not significant |  |
| CD8 Effector | H vs N | 0.772829992684448 | 1 | Not significant |  |
| CD8 Effector | L vs N | 1 | 1 | Not significant |  |
| CD8 Effector | H vs T | 0.816961321715391 | 1 | Not significant |  |
| CD8 Effector | L vs T | 0.288844366346485 | 1 | Not significant |  |
| CD8 Effector | N vs T | 1 | 1 | Not significant |  |
| CD8 Naive | H vs L | 1 | 1 | Not significant |  |
| CD8 Naive | H vs N | 0.245278116806773 | 0.966879349018126 | Not significant |  |
| CD8 Naive | L vs N | 0.54029137460742 | 0.966879349018126 | Not significant |  |
| CD8 Naive | H vs T | 0.805732790848439 | 0.966879349018126 | Not significant |  |
| CD8 Naive | L vs T | 0.69263278404196 | 0.966879349018126 | Not significant |  |
| CD8 Naive | N vs T | 0.481120001127239 | 0.966879349018126 | Not significant |  |
| CD8 StressResponse | H vs L | 0.54029137460742 | 0.893824635847767 | Not significant |  |
| CD8 StressResponse | H vs N | 0.386476230771233 | 0.893824635847767 | Not significant |  |
| CD8 StressResponse | L vs N | 1 | 1 | Not significant |  |
| CD8 StressResponse | H vs T | 0.816961321715391 | 0.980353586058469 | Not significant |  |
| CD8 StressResponse | L vs T | 0.288844366346485 | 0.893824635847767 | Not significant |  |
| CD8 StressResponse | N vs T | 0.595883090565178 | 0.893824635847767 | Not significant |  |
| CD8 Tex | H vs L | 0.54029137460742 | 0.54029137460742 | Not significant |  |
| CD8 Tex | H vs N | 0.220671361919847 | 0.54029137460742 | Not significant |  |
| CD8 Tex | L vs N | 0.479500122186953 | 0.54029137460742 | Not significant |  |
| CD8 Tex | H vs T | 0.487453290364035 | 0.54029137460742 | Not significant |  |
| CD8 Tex | L vs T | 0.288844366346485 | 0.54029137460742 | Not significant |  |
| CD8 Tex | N vs T | 0.218819415823491 | 0.54029137460742 | Not significant |  |
| CD8 Trm | H vs L | 0.54029137460742 | 0.81043706191113 | Not significant |  |
| CD8 Trm | H vs N | 0.698535358303339 | 0.838242429964006 | Not significant |  |
| CD8 Trm | L vs N | 0.54029137460742 | 0.81043706191113 | Not significant |  |
| CD8 Trm | H vs T | 0.374259319280224 | 0.81043706191113 | Not significant |  |
| CD8 Trm | L vs T | 1 | 1 | Not significant |  |
| CD8 Trm | N vs T | 0.138640633813219 | 0.81043706191113 | Not significant |  |
| CD8 pTex | H vs L | 1 | 1 | Not significant |  |
| CD8 pTex | H vs N | 1 | 1 | Not significant |  |
| CD8 pTex | L vs N | 1 | 1 | Not significant |  |
| CD8 pTex | H vs T | 0.767096868410277 | 1 | Not significant |  |
| CD8 pTex | L vs T | 1 | 1 | Not significant |  |
| CD8 pTex | N vs T | 0.746885633390364 | 1 | Not significant |  |

**Comprehensive Single-Cell Transcriptomic Profiling Reveals CD8⁺ T-Cell Differentiation Trajectories and LAG-3–Associated Exhaustion Programs in Cervical Disease Progression**

**Single-cell RNA-seq data processing and analysis**

### ****1. Data source and quality control****

Single-cell RNA-seq data were obtained from the publicly available dataset **E-MTAB-12305**, comprising a total of **10 samples**, including normal cervix (N, n = 3; N1–N3), high-grade squamous intraepithelial lesion (HSIL; H, n = 2; H1–H2), cervical tumor tissue (T, n = 4; T1–T4), and metastatic lymph node (L, n = 1; L1).
Before quality control (QC), the number of detected genes and cells in each sample were as follows: H1 (27,051 genes, 7,084 cells), H2 (28,237 genes, 13,302 cells), L1 (26,493 genes, 10,283 cells), N1 (27,916 genes, 14,764 cells), N2 (27,948 genes, 13,955 cells), N3 (25,330 genes, 11,465 cells), T1 (27,254 genes, 12,732 cells), T2 (27,713 genes, 8,818 cells), T3 (24,187 genes, 14,248 cells), and T4 (27,223 genes, 10,630 cells). After merging all samples, the combined dataset contained **32,611 genes and 117,281 cells**.

Unified quality control and filtering were applied after merging. Cells with fewer than 200 detected genes were removed, and genes expressed in fewer than three cells were excluded. Additional filtering criteria were applied to remove cells with n_genes_by_counts > 6,000 or < 200, as well as cells with a mitochondrial gene fraction (pct_counts_mt) ≥ 25%. Mitochondrial genes were defined by the “MT-” or “mt-” prefix. After QC, **102,287 cells and 30,392 genes** were retained for downstream analyses. The global UMAP embedding, sample-wise mixing, and CD8 T-cell identification are shown in **Figure18**.


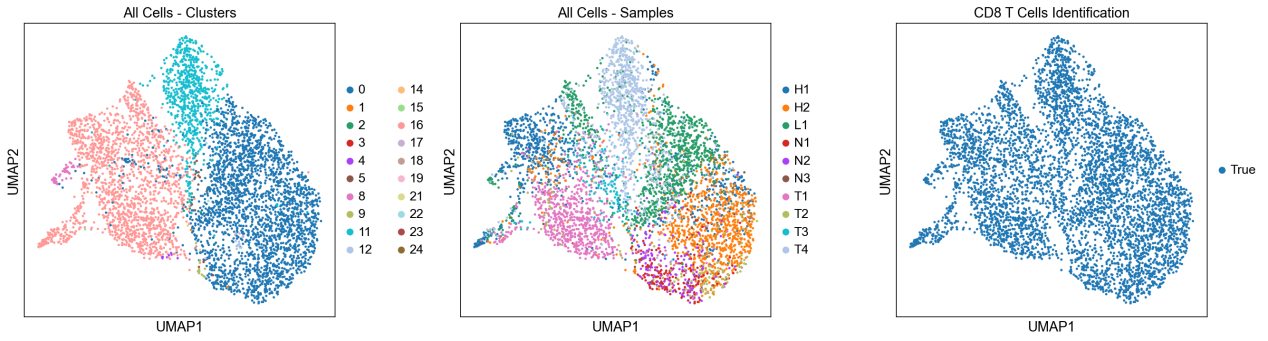


Figure18 all cells umap

### ****2. Normalization, dimensionality reduction, and clustering****

The filtered expression matrix was normalized to a total count of 10,000 per cell (target_sum = 1e4) and log-transformed using log1p. The normalized matrix was stored as a raw layer for downstream differential expression analyses. Highly variable genes (HVGs) were selected using the following thresholds: min_mean = 0.0125, max_mean = 3, and min_disp = 0.5, resulting in **3,009 HVGs** (**Figure19**).

HVGs were scaled with a maximum value of 10, followed by principal component analysis (PCA) with 50 components (n_comps = 50). Neighborhood graphs were constructed using the first 30 principal components (n_neighbors = 15, n_pcs = 30), and Uniform Manifold Approximation and Projection (UMAP) was performed for visualization. Cell clustering was conducted using the Leiden algorithm with a resolution of 0.8, yielding **26 distinct cell clusters**


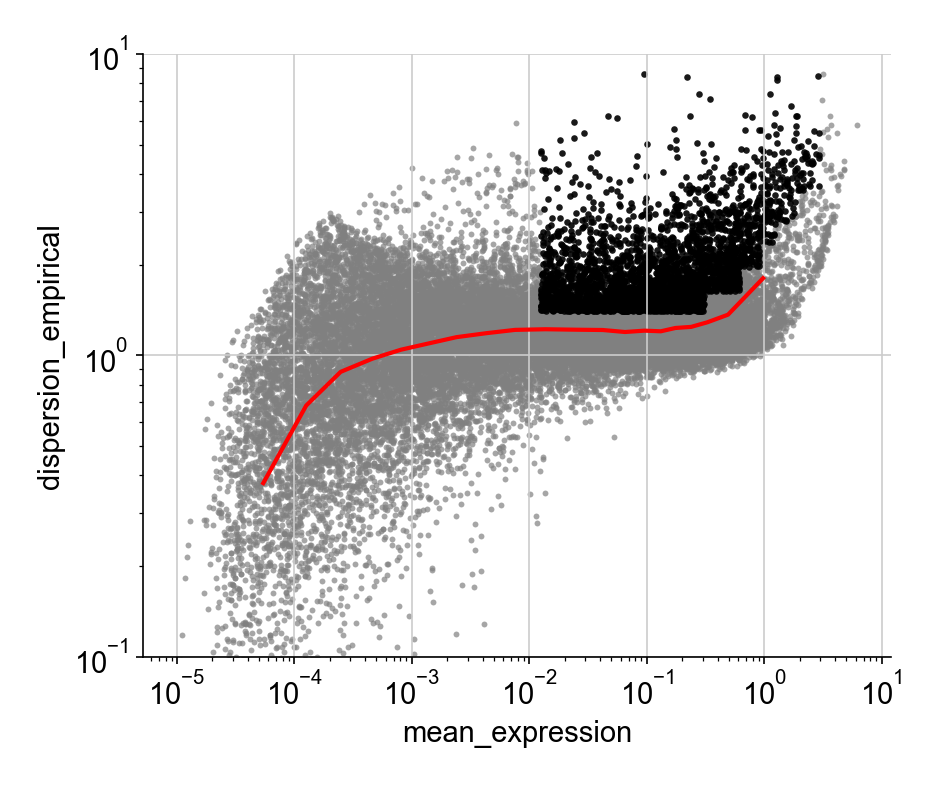


Figure 19 figure1_ordering_genes_selection

### ****3. Identification and subclustering of CD8⁺ T cells****

At the global cell level, gene set scores for canonical T-cell and CD8 markers (CD3D/CD3E/CD3G and CD8A/CD8B) were computed, and the raw expression values of these marker genes were extracted. CD8⁺ T cells were identified using a dual-threshold strategy. Specifically, the 25th percentile of the maximum expression values of CD8A/CD8B and CD3D/CD3E among cells with nonzero expression was used as the cutoff. Cells simultaneously exceeding both thresholds (CD8_expression > cutoff and T_cell_expression > cutoff) were classified as CD8⁺ T cells. If fewer than 100 cells were identified, the criterion was relaxed to include cells with CD8 expression above the 70th percentile of all cells.

Using this approach, **5,060 CD8⁺ T cells** (4.95% of all cells) were identified. Their distribution in the global UMAP embedding is shown in the right panel of **Figure18** .

The CD8⁺ T-cell subset was further subjected to neighborhood graph construction and secondary clustering using the Leiden algorithm (resolution = 0.6), resulting in **10 CD8-specific clusters**. Subcluster annotation was performed based on module scores for four functional states—Naive, Memory, Effector, and Exhausted—using established marker gene sets (CCR7/SELL/TCF7; IL7R/EOMES; GZMB/GZMA/PRF1; PDCD1/LAG3/TIGIT/HAVCR2/TOX). The annotated CD8⁺ T-cell subpopulations included Effector (3,921 cells, 77.5%), Memory (756 cells, 14.9%), Naive (198 cells, 3.9%), and Exhausted (185 cells, 3.7%). UMAP embedding of CD8⁺ T cells revealed a pronounced continuum of cellular states, with early pseudotime regions enriched for memory- and stem-like gene programs (TCF7, CCR7, IL7R, SELL), whereas later regions were characterized by upregulation of the exhaustion marker LAG-3, accompanied by increased expression of cytotoxic (GZMB, PRF1, GNLY) and proliferative (MKI67) signatures. The underlying CD8⁺ subcluster architecture, pseudotime mapping, and spatial distribution of key functional markers are summarized in Supplementary **Figure 20**.Dotplot analysis delineates the orchestrated progression of key functional programs along the CD8⁺ T-cell differentiation continuum. Upregulation of LAG-3 demarcates a terminal, fate-restricted state, whereas markers of exhaustion, cytotoxicity, proliferation, and memory/homing emerge as downstream functional readouts of this regulatory checkpoint. Subcluster-defining marker expression is visualized in Supplementary **Figure 21**.


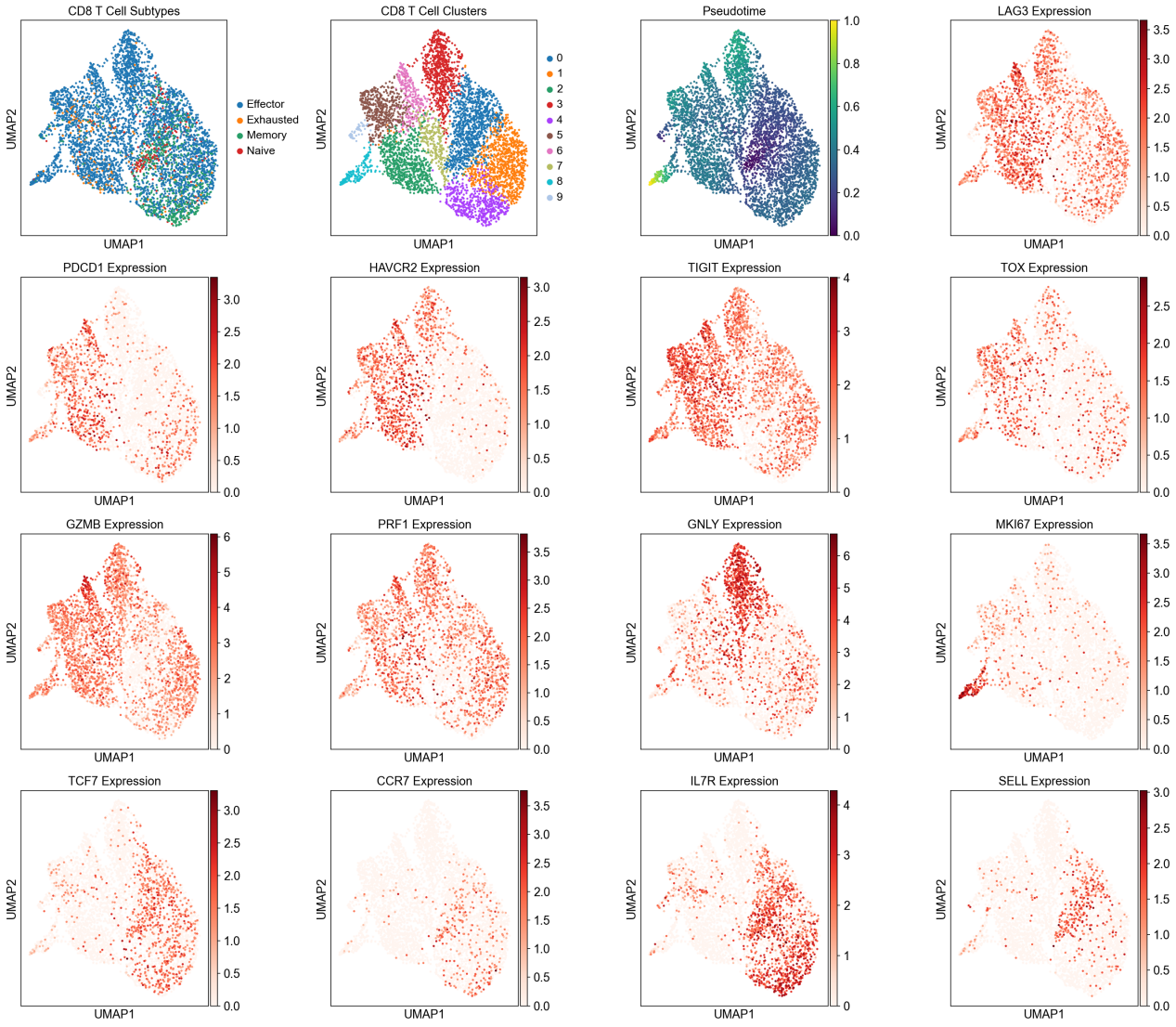


Figure 20 CD8 analysis Subclustering and functional state annotation of CD8⁺ T cells


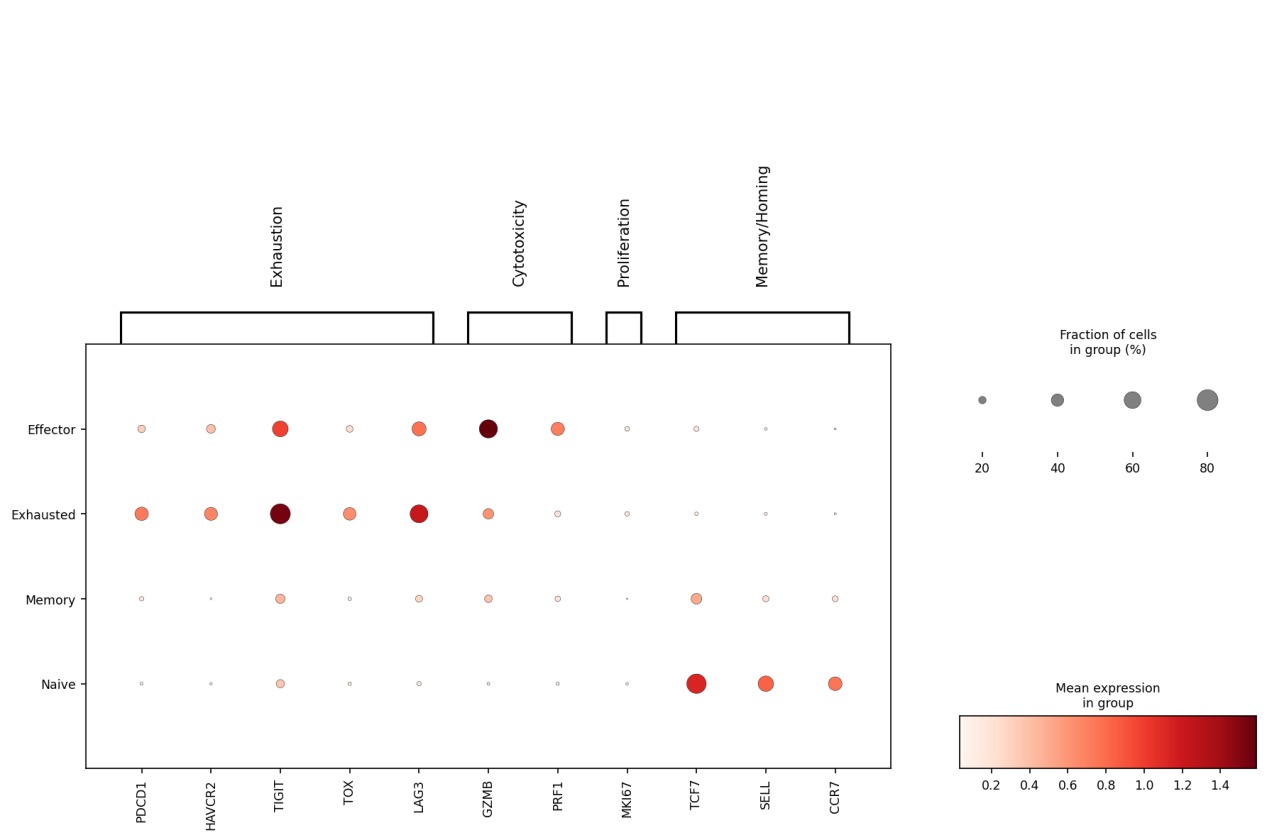


Figure 21 marker genes dotplot

### ****4.Pseudotime trajectory inference and LAG-3–centered exhaustion program along the CD8⁺ T-cell differentiation axis****

To reconstruct CD8⁺ T-cell state transitions, diffusion maps were computed within the CD8⁺ T-cell subset, and diffusion pseudotime (DPT) analysis was applied to infer a continuous developmental trajectory. This analysis yielded pseudotime values ranging from 0 to 1 (median = 0.324), capturing gradual transitions rather than discrete cell states. To define the global topology of state progression, partition-based graph abstraction (PAGA) was constructed based on the 10 CD8-specific clusters, resulting in a 10 × 10 connectivity matrix with 86 nonzero edges. The principal trajectory inferred from the PAGA graph was overlaid onto the diffusion map for visualization (**Figure 22**; **Figure 23**).

Importantly, trajectories colored by sample origin demonstrated contributions from all 10 samples (e.g., L1 = 1,008 cells; H2 = 943; H1 = 882; T4 = 826; T1 = 744), indicating that the inferred developmental axis was not driven by a single sample or batch effect but reflected a shared biological program (**Figure24**).

Within this reconstructed differentiation framework, **LAG-3 was explicitly positioned as a regulatory checkpoint associated with late-stage CD8⁺ T-cell dysfunction**, rather than being treated as one of multiple equivalent exhaustion markers. Accordingly, LAG3 expression was prioritized as the primary variable to stratify cells along the trajectory. Alternative gene symbols (LAG3/Lag3/CD223) were considered where applicable. In rare cases where LAG3 expression was absent, the first available marker among PDCD1, CTLA4, TIGIT, HAVCR2, or TOX was used as a conservative surrogate, and its expression was recorded as LAG3_expression to preserve continuity in downstream analyses.

Using the median single-cell expression value as the threshold, CD8⁺ T cells were stratified into LAG3-high and LAG3-low groups, yielding 2,137 LAG3-high cells (42.2%) and 2,923 LAG3-low cells (57.8%). The median LAG3_expression was 0, with a 95th percentile value of 2.27. This stratification enabled interrogation of how LAG-3–associated states align with progression along the inferred differentiation axis.

Differential expression analysis between LAG3-high and LAG3-low cells identified 388 significantly differentially expressed genes (|logFC| > 0.5, FDR < 0.05), including 374 upregulated and 14 downregulated genes. Notably, genes associated with cytotoxic effector function and exhaustion (e.g., **GZMB, CXCL13, ENTPD1, CTLA4**) were enriched in the LAG3-high group, whereas genes linked to memory potential and early differentiation states (e.g., **IL7R, TCF7, KLF2, GZMK**) were preferentially expressed in LAG3-low cells. Pathway enrichment analysis further revealed that genes upregulated in LAG3-high cells were significantly enriched for immune activation and antigen processing pathways (e.g., **Proteasome pathway; adjusted P = 8.18 × 10⁻⁹**).

Crucially, markers related to memory maintenance (TCF7, CCR7, IL7R, SELL), cytotoxicity (GZMB, PRF1, GNLY), proliferation (MKI67), and exhaustion (PDCD1, HAVCR2, TIGIT, TOX) were not interpreted as independent regulators. Instead, their expression dynamics were analyzed as **phenotypic and functional readouts downstream of the LAG-3–associated regulatory state**, positioned along the same pseudotime-defined differentiation axis. Consistent with this hierarchical model, LAG3-high cells localized predominantly to later pseudotime regions, where exhaustion- and effector-associated programs were enhanced, whereas LAG3-low cells were enriched at earlier stages characterized by memory- and stemness-associated features.

The spatial localization of LAG3-high versus LAG3-low cells in UMAP space, together with expression distributions, volcano plots, top DEG heatmaps, and subpopulation composition analyses, are shown in **Figure25**. LAG3 group labels were further projected onto the inferred trajectory (**Figure26**), complemented by trajectory-based gene expression projections (**Figure27**) and pseudotime heatmaps (**Figure28**). Collectively, these analyses place **LAG-3 at a late differentiation checkpoint that constrains the transition from memory-like to terminal effector/exhausted CD8⁺ T-cell states**, providing a coherent framework that links transcriptional dynamics, trajectory structure, and functional readouts.


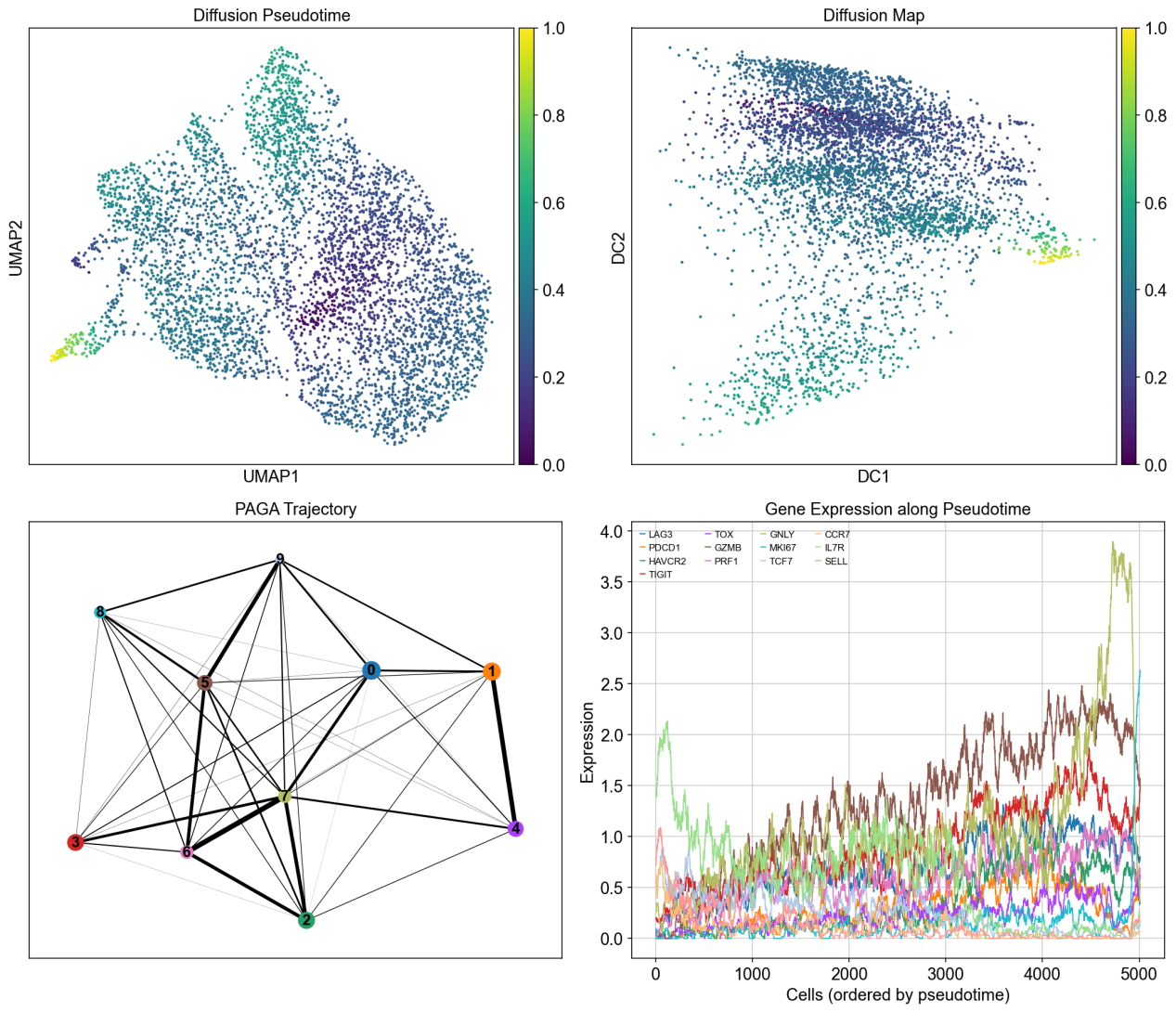


Figure 22 trajectory analysis


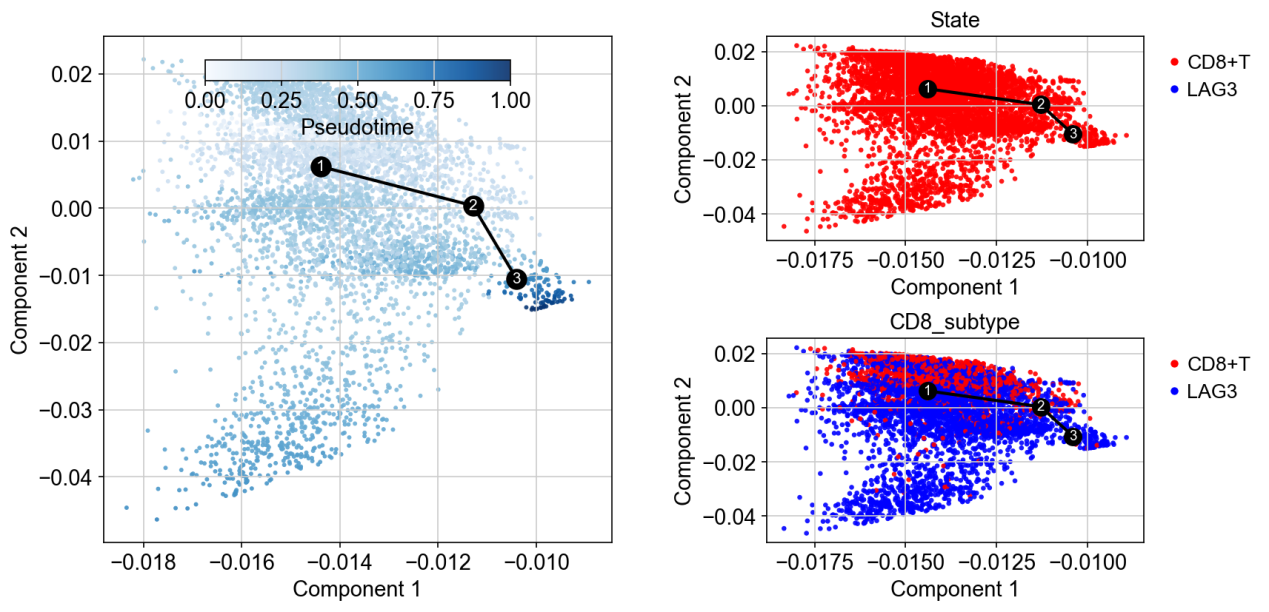


Figure23 development trajectory


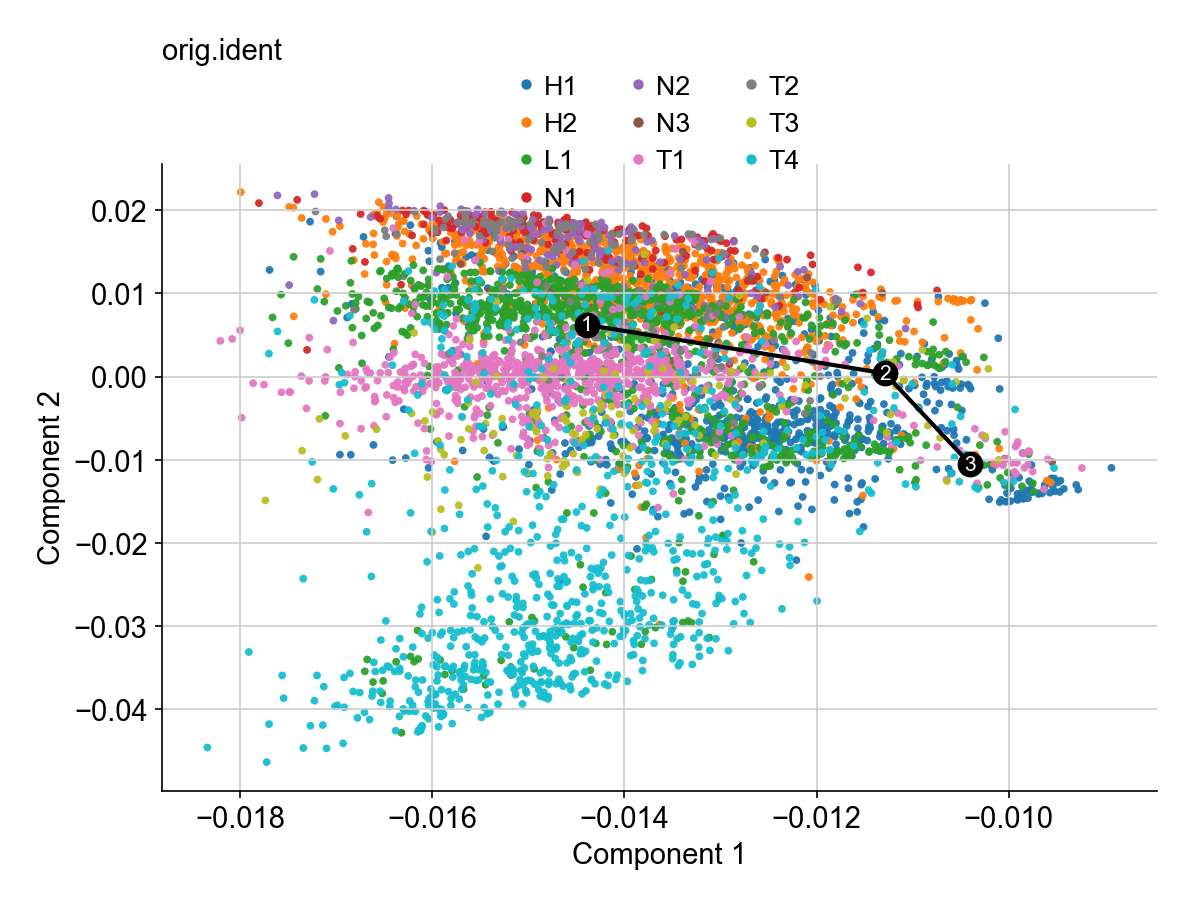


Figure24 development trajectory sample


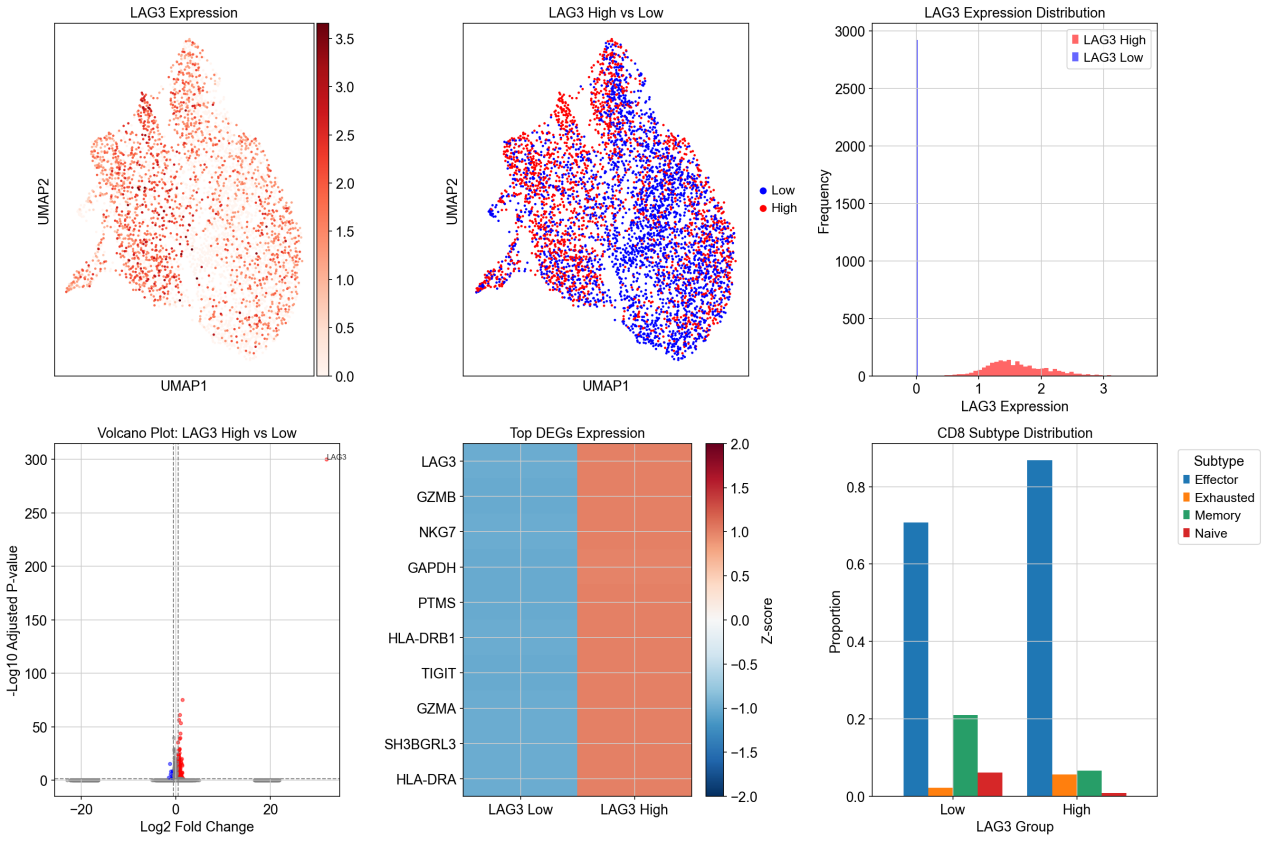


Figure25 lag3 analysis.


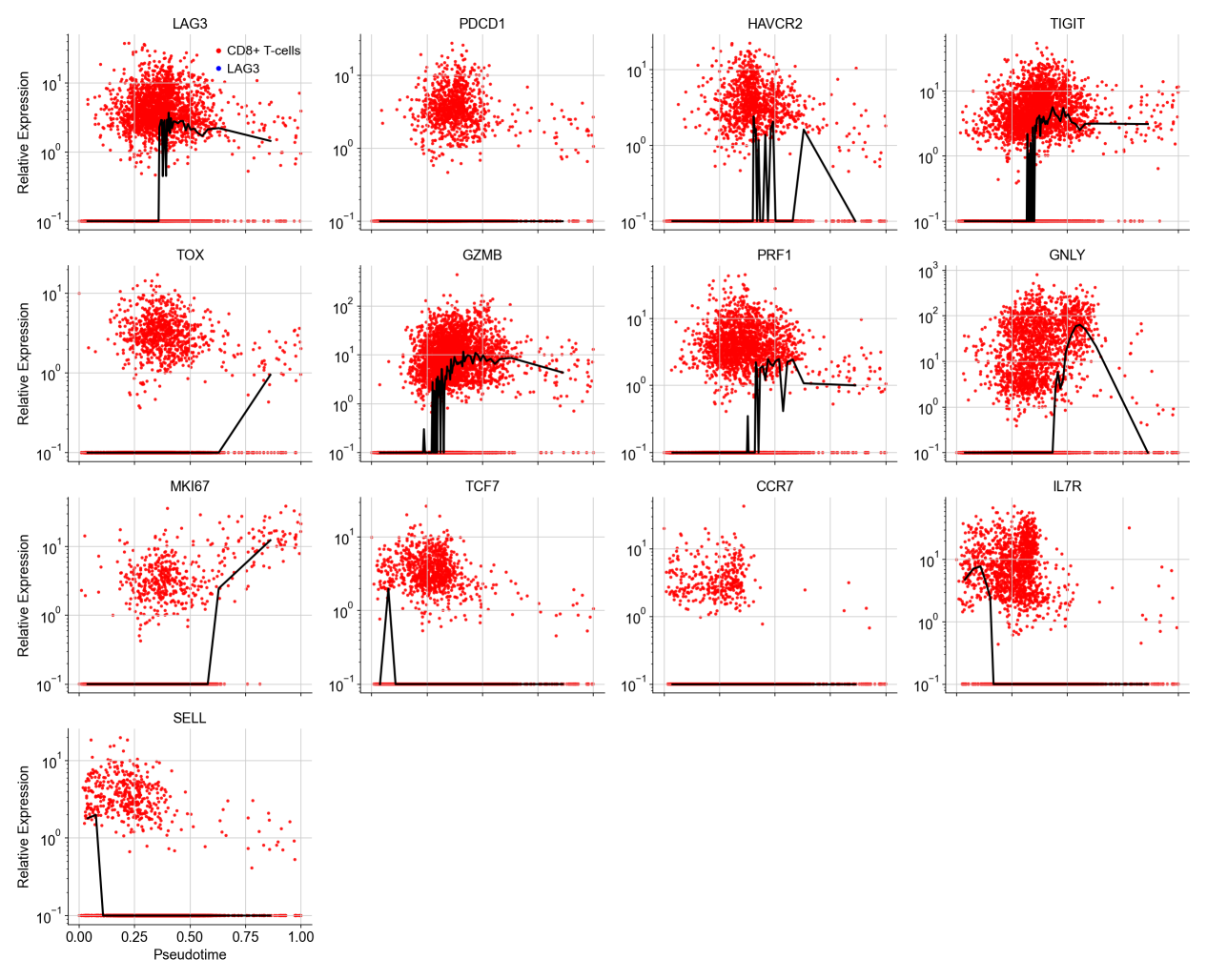
Figure26 development trajectory pseudotime scatter


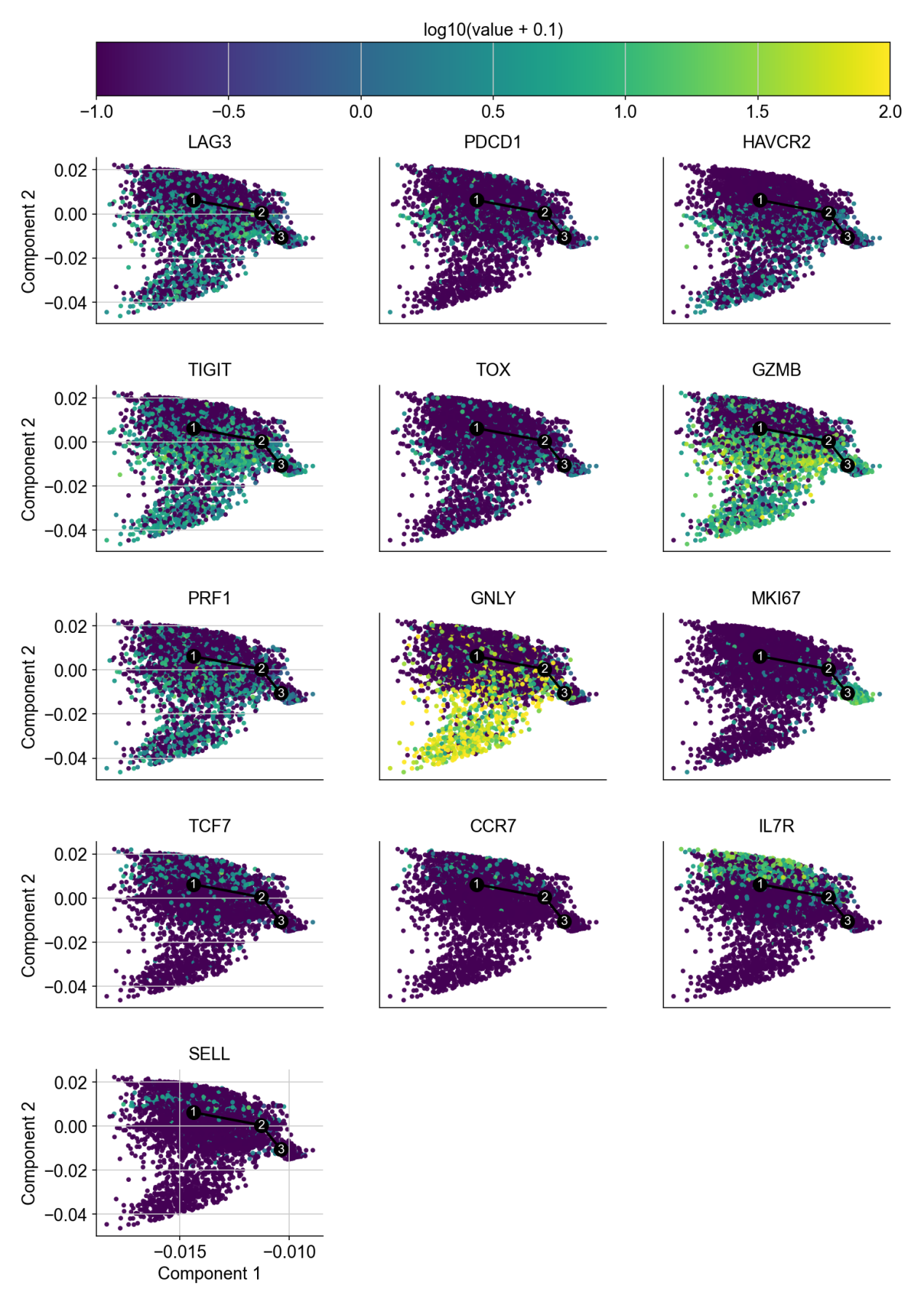
Figure27 development trajectory gene expression


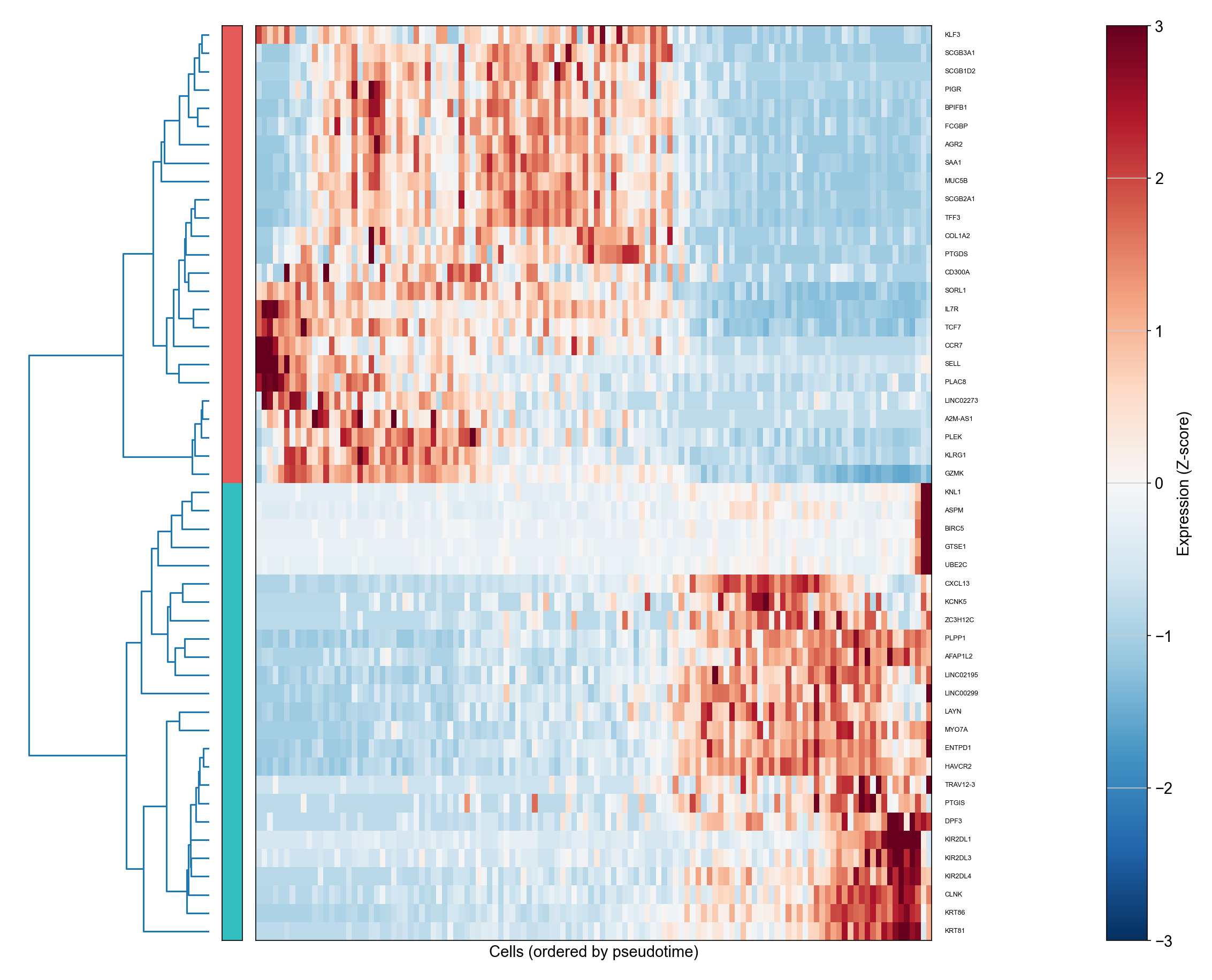
Figure28 pseudotime gene heatmap

Table 6 Association between LAG-3 expression and clinicopathological characteristics and survival in cervical cancer

| independent variable | LAG3阳性% | | c2/Z | P |
| --- | --- | --- | --- | --- |
|  | ≤50% | >50% |  |  |
| Age | 52(43.5,58) | 48(41.75,57.25) | -0.432 | 0.666 |
| Histological classification |  |  | 1.183 | 1 |
| Adenosquamous carcinoma | 0(0.00%) | 1(100.00%) |  |  |
| Squamous cell carcinoma | 5(9.80%) | 46(90.20%) |  |  |
| Adenocarcinoma | 1(12.50%) | 7(87.50%) |  |  |
| Histological differentiation |  |  | 7.771 | 0.014 |
| poorly differentiated | 0(0.00%) | 19(100.00%) |  |  |
| Moderately differentiated | 3(8.80%) | 31(91.20%) |  |  |
| Well-differentiated | 3(42.90%) | 4(57.10%) |  |  |
| FIGO stage |  |  | 24.745 | <0.01 |
| ⅠA | 5(100.00%) | 0(0.00%) |  |  |
| IB | 1(7.70%) | 12(92.30%) |  |  |
| ⅡA1 | 0(0.00%) | 20(100.00%) |  |  |
| ⅡA2 | 0(0.00%) | 12(100.00%) |  |  |
| ⅢC1 | 0(0.00%) | 10(100.00%) |  |  |
| Lymph node metastasis |  |  | 1.333 | 0.577 |
| YES | 6(12.00%) | 44(88.00%) |  |  |
| NO | 0(0.00%) | 10(100.00%) |  |  |
| Lymphovascular and perineural invasion |  |  | 0.625 | 0.429 |
| YES | 5(13.90%) | 31(86.10%) |  |  |
| NO | 1(4.20%) | 23(95.80%) |  |  |
| Five-year overall survival time (1825 days) | 494(422,648.25) | 396.5(274,584.25) | -1.244 | 0.213 |
| vital status |  |  |  |  |
| YES | 6(11.50%) | 46(88.50%) | 0.144 | 0.704 |
| NO | 0(0.00%) | 8(100.00%) |  |  |


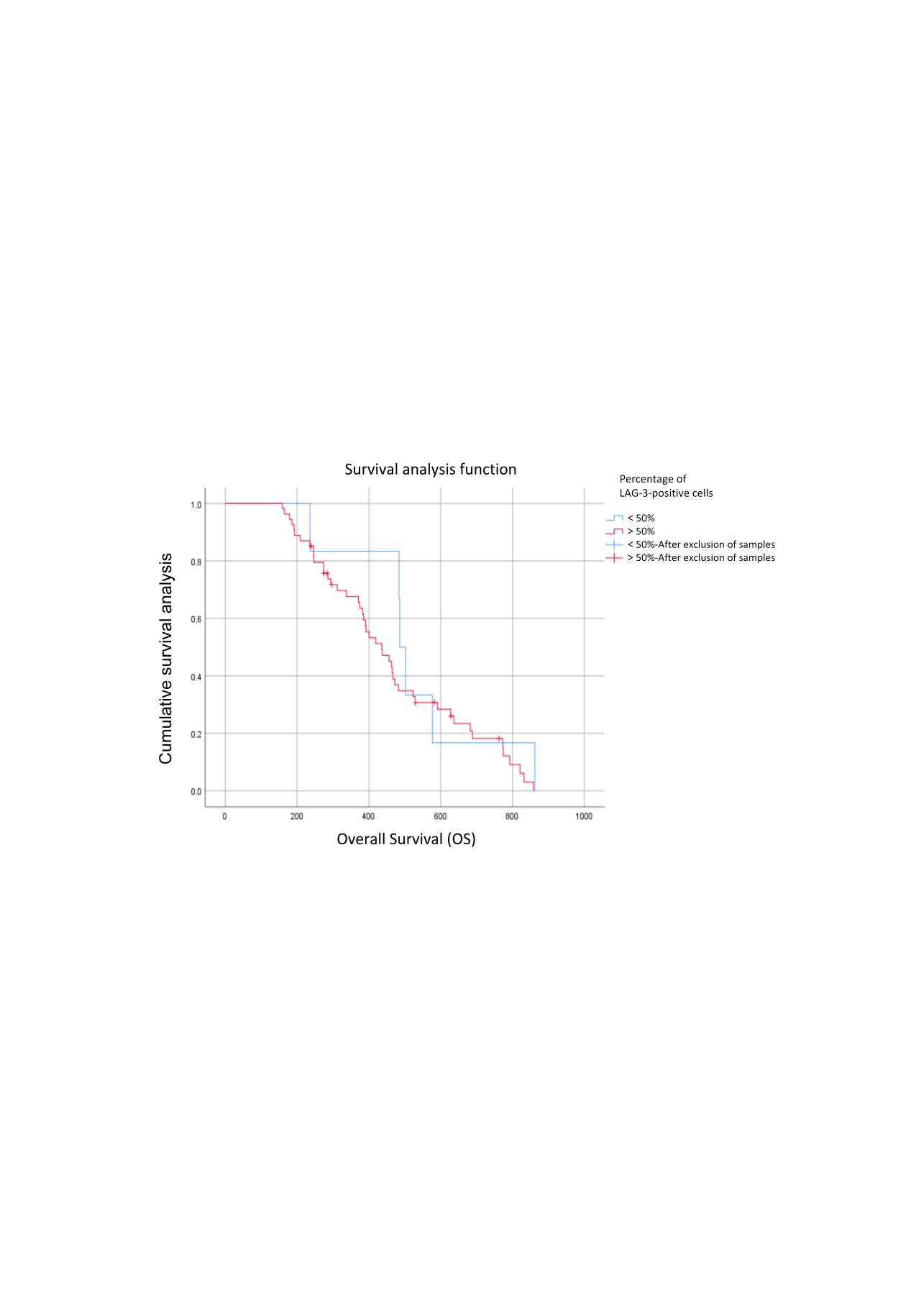


Figure29 Kaplan–Meier Survival Analysis Stratified by LAG-3 Expression in Cervical Cancer Patients
