# Supplementary material for: LAG-3–associated CD8+ T-cell dysfunction in the cervical cancer tumor microenvironment
Source: Front Immunol. 2026 Jan 28;17:1750726. doi: 10.3389/fimmu.2026.1750726 (PMC12891123; doi:10.3389/fimmu.2026.1750726)
Supplement: Supplementary file 2 [file Presentation1.pptx]

## Slide 1
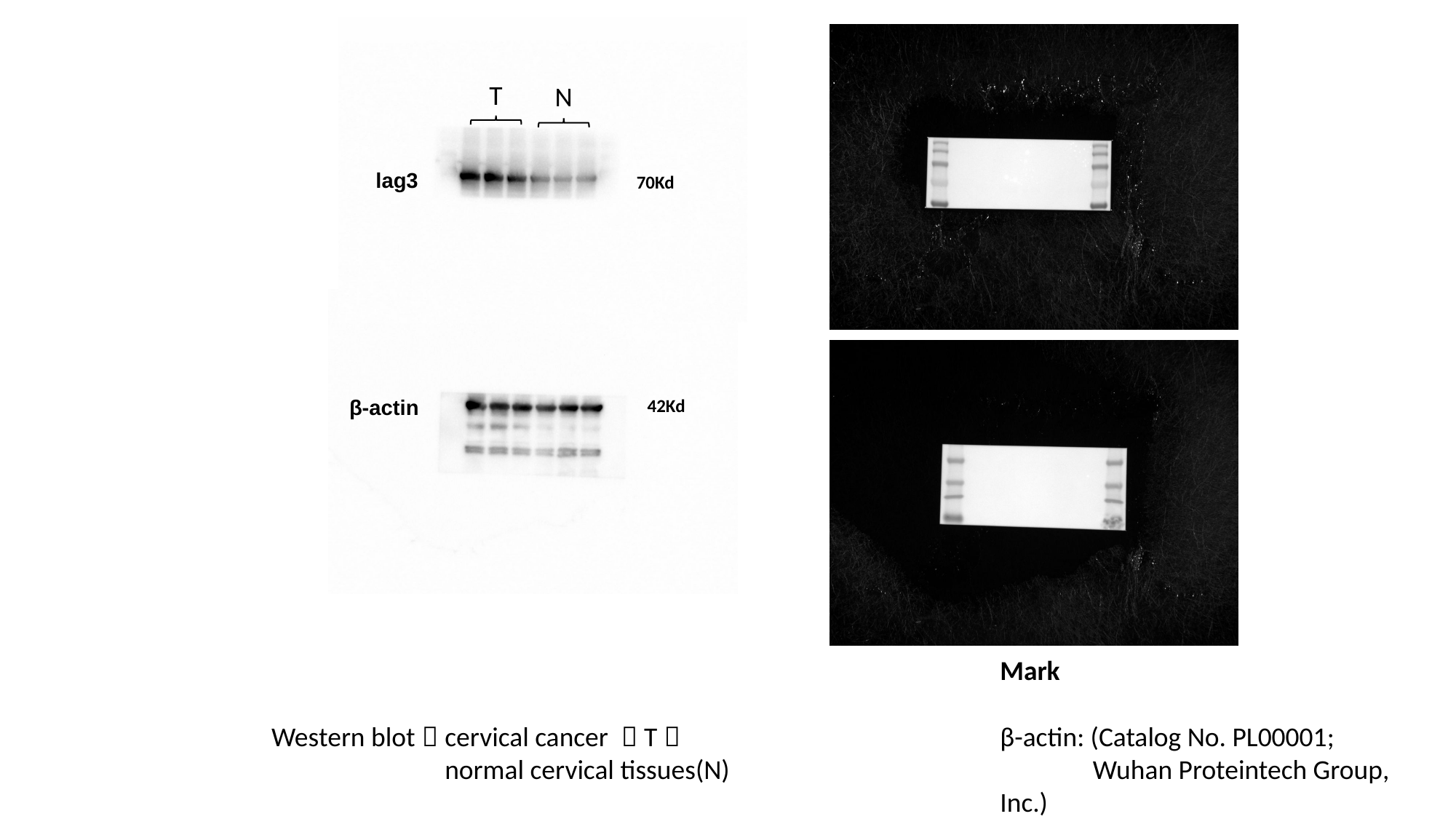

T
N
lag3
70Kd
β-actin
42Kd
Mark
Western blot：cervical cancer （T）
 normal cervical tissues(N)
β-actin: (Catalog No. PL00001;
 Wuhan Proteintech Group, Inc.)
